# Synthesis and Cytotoxicity of *N*-Substituted Dibenzo[*a*,*j*]xanthene-3,11-dicarboxamide Derivatives

**DOI:** 10.3390/molecules22040517

**Published:** 2017-03-23

**Authors:** Yongbin Song, Yihui Yang, Lijun Wu, Naiwei Dong, Shang Gao, Hongrui Ji, Xia Du, Bo Liu, Guoyou Chen

**Affiliations:** 1 School of Chemical and Environmental Engineering, Harbin University of Science and Technology, Harbin 150040, China; songyongbin1981@hrbust.edu.cn (Y.S.); sy23818@163.com (H.J.); yazx23@126.com (X.D.); lb765812@126.com (B.L.); 2 College of Pharmacy, Harbin Medical University, Harbin 150081, China; wkhg1823@sohu.com (L.W.); cvrf568@163.com (N.D.); gtm8745@126.com (S.G.); 3 College of Pharmacy, Harbin Medical University Daqing Campus, Daqing 163319, China; dmf8198@sina.com

**Keywords:** synthesis design, cytotoxicity, dibenzo[*a*,*j*]xanthenes, NMR spectroscopy

## Abstract

In order to study the structure-activity relationships of xanthene derivatives, four series of *N*-substituted 14-aryl-14*H*-dibenzo[*a*,*j*]xanthene-3,11-dicarboxamide derivatives were synthesized. The structures of all compounds were identified by ^1^H-NMR, HR-MS and IR spectra, in which compounds **6a**–**h** were further identified by ^13^C-NMR spectra. The in vitro antitumor activity of the synthesized compounds was tested by MTT assay. Most of them displayed strong inhibitory activity on human hepatocellular carcinoma cell lines (SK-HEP-1, HepG2 and SMMC-7721 cells) and acute promyelocytic leukemia NB4 cells. Compounds **6c**–**6e** exhibited significant inhibitory activity against NB4 cells with IC_50_ values of 0.52 μM and 0.76 μM, respectively, much lower than 5.31 μM of the positive control As_2_O_3_.

## 1. Introduction

The benzoxanthenes have attracted considerable interest due to their biological and pharmacological activities such as antiviral [1], antibacterial [2], anti-inflammation [3], antitumor [4] and other usages in photodynamic therapy [5], and the antagonizing paralysis induced by zoxazolamine [6].

**Figure 1:**
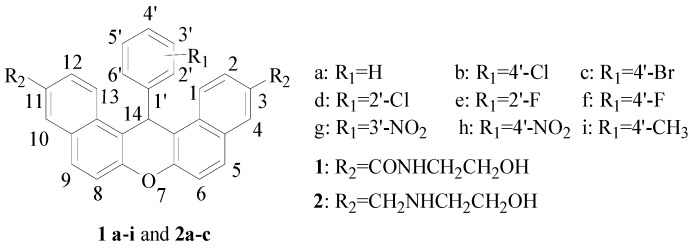
Structures of compounds **1a**–**i** and **2a**–**c**.

In view of the great importance of benzoxanthenes, the preparation of which has been a hot research topic, there have been many research reports in recent years [7,8,9,10,11]. So far the synthesis of dibenzo[*a*,*j*]xanthene has been mostly focused on the modification of the 14-position of the molecule, with other positions rarely reported in the literature, and especially the 3 and the 11 positions. In our previous work, we synthesized two series of dibenzo[*a*,*j*]xanthene-3,11-substituted compounds bearing a 2-hydroxyethyl group on the nitrogen atom (**1a**–**i** and **2a**–**c**, Figure 1) [12]. The results of in vitro antitumor activity experiments revealed that compounds **1a**–**i** and **2a**–**c** exhibit remarkable inhibitory activity toward a wide range of human tumor cell lines. Furthermore, the amide derivatives **1a**–**c** exhibit a stronger inhibitory effect than the amine derivatives **2a**–**c** on tumor cell lines, implying that the amide group of dibenzo[*a*,*j*]xanthene derivatives is more critical than the amine group for improving the inhibitory activity toward tumor cells.

**Figure 2:**
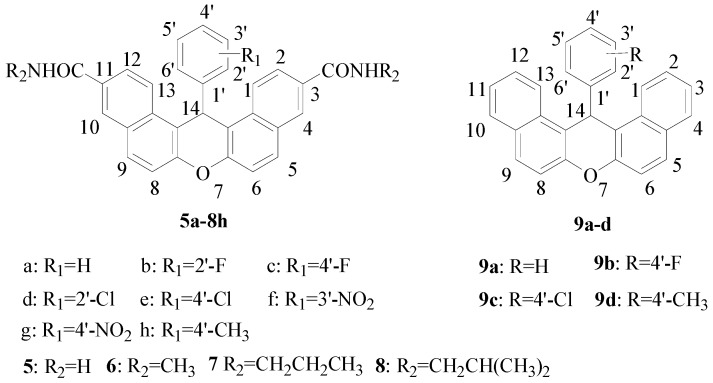
Structures of compounds **5a**–**9d**.

These results prompted us to synthesize new analogues of compounds **1a**–**i** containing different substituted amide groups, in order to clarify the structure-activity relationships (SARs) of xanthene analogues. Herein, we first describe the synthesis of derivatives **5a**–**h**, **6a**–**h**, **7a**–**h** and **8a**–**h** (Figure 2), and then report the screening results on their cytotoxicity against four cancer cell lines.

## 2. Results

### 2.1. Chemistry

**Scheme 1:**
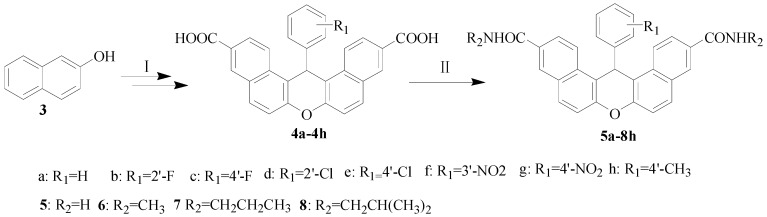
Synthesis of compounds **5a**–**h**, **6a**–**h**, **7a**–**h** and **8a**–**h**. *Reagents and conditions*: (I) See References [12,13,14]; (II) 1. Compounds **5a**–**5h**: NH_3_, CHCl_3_, r.t., 2–3 h; 2. Compounds **6a**–**6h**: CH_3_NH_2_, CHCl_3_, r.t., 2–3 h; 3. Compounds **7a**–**7h**: CH_3_CH_2_CH_2_NH_2_, CHCl_3_, 2–3 h, r.t.; 4. Compounds **8a**–**8h**: (CH_3_)_2_CHCH_2_NH_2_, CHCl_3_, r.t., 2–3 h.

The synthetic route for the carboxamide derivatives **5a**–**h**, **6a**–**h**, **7a**–**h** and **8a**–**h** is outlined in Scheme 1.

*14-Phenyl-14H-dibenzo[a*,*j]xanthene-3*,*11-dicarboxamide* (**5a**): White solid. Yield 84.2%. m.p. 229–231 °C. ^1^H-NMR (400 MHz, DMSO-*d*_6_) δ (in ppm): 8.74 (d, *J* = 8.9 Hz, 2H, H-1, 13), 8.49 (s, 2H, H-4, 10), 8.10 (s, 2H, CONH × 2), 8.04 (overlapping t, *J* = 8.9 Hz, 4H), 7.72–7.57 (m, 4H), 7.44 (s, 2H, CONH × 2), 7.15 (t, *J* = 7.6 Hz, 2H, H-3′, 5′), 6.98 (t, *J* = 7.2 Hz, 1H, H-4′), 6.79 (s, 1H, H-14). IR (KBr) ν: 3422, 1655, 1623, 1595, 1466, 1396, 1244, 1081 cm^−1^. HR-MS (ESI) calcd for C_29_H_21_N_2_O_3_ [M + H]^+^ 445.1552, found 445.1559.

*14-(2-Fluorophenyl)-14H-dibenzo[a*,*j]xanthene-3*,*11-dicarboxamide* (**5b**): White solid. Yield 83.7%. m.p. 229–230 °C. ^1^H-NMR (400 MHz, DMSO-*d*_6_) δ (in ppm): 8.51 (d, *J* = 1.5 Hz, 2H, H-4, 10), 8.47 (d, *J* = 9.0 Hz, 2H, H-1, 13), 8.10 (s, 2H, CONH × 2), 8.07(dd, *J* = 9.0, 1.7 Hz, 2H, H-2, 12), 8.06 (d, *J* = 9.0 Hz, 2H, H-5, 9), 7.63 (d, *J* = 8.9 Hz, 2H, H-6, 8), 7.62–7.57 (m, 1H, H-6′), 7.45 (s, 2H, CONH × 2), 7.14–6.97 (m, 3H, H-3′, 4′, 5′), 6.90 (s, 1H, H-14). IR (KBr) ν: 3409, 1658, 1624, 1595, 1467, 1397, 1256, 1245 cm^−1^. HR-MS (ESI) calcd for C_29_H_20_FN_2_O_3_ [M + H]^+^ 463.1458, found 463.1455.

*14-(4-Fluorophenyl)-14H-dibenzo[a*,*j]xanthene-3*,*11-dicarboxamide* (**5c**): White solid. Yield 87.9%. m.p. 241–243 °C. ^1^H-NMR (400 MHz, DMSO-*d*_6_) δ (in ppm): 8.74 (d, *J* = 9.0 Hz, 2H, H-1, 13), 8.51 (d, *J* = 1.6 Hz, 2H, H-4, 10), 8.12 (s, 2H, CONH × 2), 8.06 (dd, *J* = 9.0, 1.7 Hz, 2H, H-2, 12), 8.04 (d, *J* = 8.9 Hz, 2H, H-5, 9), 7.72–7.62 (m, 2H, H-2′, 6′), 7.63 (d, *J* = 8.9 Hz, 2H, H-6, 8), 7.45 (s, 2H, CONH × 2), 6.99 (t, *J* = 8.9 Hz, 2H, H-3′, 5′), 6.82 (s, 1H, H-14). IR (KBr) ν: 3394, 3157, 3049, 1655, 1623, 1506, 1466, 1401, 1245 cm^−1^. HR-MS (ESI) calcd for C_29_H_20_FN_2_O_3_ [M + H]^+^ 463.1458, found 463.1469.

*14-(2-Chlorophenyl)-14H-dibenzo[a*,*j]xanthene-3*,*11-dicarboxamide* (**5d**): White solid. Yield 82.5%. m.p. 235–236 °C. ^1^H-NMR (400 MHz, DMSO-*d*_6_) δ (in ppm): 8.65 (d, *J* = 9.0 Hz, 2H, H-1, 13), 8.51 (d, *J* = 1.5 Hz, 2H, H-4, 10), 8.10 (s, 2H, CONH × 2), 8.08 (dd, *J* = 9.0, 1.7 Hz, 2H, H-2, 12), 8.07 (d, *J* = 9.0 Hz, 2H, H-5, 9), 7.64 (d, *J* = 8.9 Hz, 2H, H-6, 8), 7.55 (d, *J* = 7.4 Hz, 1H, H-6′), 7.45 (s, 2H, CONH × 2), 7.34 (dd, *J* = 8.0, 1.1 Hz, 1H, H-3′), 7.15 (t, *J* = 7.0 Hz, 1H, H-5′), 7.07 (td, *J* = 7.8, 1.6 Hz, 1H, H-4′), 6.88 (s, 1H, H-14). IR (KBr) ν: 3377, 3192, 1657, 1623, 1594, 1467, 1396, 1254 cm^−1^. HR-MS (ESI) calcd for C_29_H_20_ClN_2_O_3_ [M + H]^+^ 479.1162, found 479.1170.

*14-(4-Chlorophenyl)-14H-dibenzo[a*,*j]xanthene-3*,*11-dicarboxamide* (**5e**): White solid. Yield 82.3%. m.p. 231–233 °C. ^1^H-NMR (400 MHz, DMSO-*d*_6_) δ (in ppm): 8.72 (d, *J* = 8.9 Hz, 2H, H-1, 13), 8.50 (d, *J* = 1.6 Hz, 2H, H-4, 10), 8.11 (s, 2H, CONH × 2), 8.06 (dd, *J* = 8.9, 1.6 Hz, 2H, H-2, 12), 8.05 (d, *J* = 8.9 Hz, 2H, H-5, 9), 7.65 (d, *J* = 8.6 Hz, 2H, H-2′, 6′), 7.64 (d, *J* = 8.9 Hz, 2H, H-6, 8), 7.45 (s, 2H, CONH × 2), 7.23 (d, *J* = 8.6 Hz, 2H, H-3′, 5′), 6.82 (s, 1H, H-14). IR (KBr) ν: 3396, 3185, 1656, 1623, 1593, 1466, 1398, 1253 cm^−1^. HR-MS (ESI) calcd for C_29_H_20_ClN_2_O_3_ [M + H]^+^ 479.1162, found 479.1168.

*14-(3-Nitrophenyl)-14H-dibenzo[a*,*j]xanthene-3*,*11-dicarboxamide* (**5f**): White solid. Yield 81.8%. m.p. 225–227 °C. ^1^H-NMR (400 MHz, DMSO-*d*_6_) δ (in ppm): 8.81 (d, *J* = 9.0 Hz, 2H, H-1, 13), 8.56 (t, *J* = 1.9 Hz, 1H, H-2′), 8.51 (d, *J* = 1.6 Hz, 2H, H-4, 10), 8.12 (s, 2H, CONH × 2), 8.11–8.06 (m, 5H), 7.87 (br.d, *J* = 7.6 Hz, 1H, H-4′), 7.68 (d, *J* = 8.9 Hz, 2H, H-6, 8), 7.48 (t, *J* = 8.1 Hz, 1H, H-5′), 7.46 (s, 2H, CONH × 2), 7.04 (s, 1H, H-14). IR (KBr) ν: 3434, 3186, 1664, 1622, 1594, 1521, 1465, 1397, 1348, 1253 cm^−1^. HR-MS (ESI) calcd for C_29_H_20_N_3_O_5_ [M + H]^+^ 490.1403, found 490.1411.

*14-(4-Nitrophenyl)-14H-dibenzo[a*,*j]xanthene-3*,*11-dicarboxamide* (**5g**): White solid. Yield 83.7%. m.p. 238–240 °C. ^1^H-NMR (400 MHz, DMSO-*d*_6_) δ (in ppm): 8.75 (d, *J* = 9.0 Hz, 2H, H-1, 13), 8.51 (d, *J* = 1.5 Hz, 2H, H-4, 10), 8.12 (s, 2H, CONH × 2), 8.10-8.02 (m, 6H), 7.93 (d, *J* = 8.9 Hz, 2H, H-2′, 6′), 7.67 (d, *J* = 8.9 Hz, 2H, H-6, 8), 7.46 (s, 2H, CONH × 2), 7.00 (s, 1H, H-14). IR (KBr) ν: 3382, 3191, 1661, 1623, 1607, 1594, 1516, 1466, 1397, 1345, 1254 cm^−1^. HR-MS (ESI) calcd for C_29_H_20_N_3_O_5_ [M + H]^+^ 490.1403, found 490.1398.

*14-(4-Methylphenyl)-14H-dibenzo[a*,*j]xanthene-3*,*11-dicarboxamide* (**5h**): White solid. Yield 84.2%. m.p. 229–231 °C. ^1^H-NMR (400 MHz, DMSO-*d*_6_) δ (in ppm): 8.72 (d, *J* = 9.0 Hz, 2H, H-1, 13), 8.50 (d, *J* = 1.4 Hz, 2H, H-4, 10), 8.12 (s, 2H, CONH × 2), 8.05 (dd, *J* = 9.0, 1.6 Hz, 2H, H-2, 12), 8.02 (d, *J* = 9.0 Hz, 2H, H-5, 9), 7.62 (d, *J* = 8.9 Hz, 2H, H-6, 8), 7.50 (d, *J* = 8.1 Hz, 2H, H-2′, 6′), 7.45 (s, 2H, CONH × 2), 6.95 (d, *J* = 8.0 Hz, 2H, H-3′, 5′), 6.74 (s, 1H, H-14), 2.05 (s, 3H, CH_3_). IR (KBr) ν: 3398, 3193, 1656, 1623, 1594, 1466, 1398, 1255, 1244 cm^−1^. HR-MS (ESI) calcd for C_30_H_23_N_2_O_3_ [M + H]^+^ 459.1709, found 459.1701.

*N^3^_,_N^11^-Dimethyl-14-phenyl-14H-dibenzo[a*,*j]xanthene-3*,*11-dicarboxamide* (**6a**): White solid. Yield 87.1%. m.p. >300 °C. ^1^H-NMR (300 MHz, DMSO-*d*_6_) δ (in ppm): 8.74 (d, *J* = 9.0 Hz, 2H, H-1, 13), 8.63 (q, *J* = 4.5 Hz, 2H, CONH × 2), 8.45 (br.s, 2H, H-4, 10), 8.03 (overlapping d, *J* = 8.9 Hz, 4H), 7.62 (overlapping d, *J* = 8.7 Hz, 4H), 7.13 (t, *J* = 7.6 Hz, 2H, H-3′, 5′), 6.96 (t, *J* = 7.3 Hz, 1H, H-4′), 6.77 (s, 1H, H-14), 2.82 (d, *J* = 4.4 Hz, 6H, CH_3_ × 2). ^13^C-NMR (75 MHz, DMSO-*d*_6_) δ (in ppm): 166.9, 149.4, 145.8, 132.6, 131.0, 130.6, 130.4, 129.0, 128.6, 128.4, 126.9, 125.4, 124.1, 118.9, 118.0, 36.9, 26.8. IR (KBr) ν: 3434, 1638, 1620, 1546, 1464, 1400, 1251 cm^−1^. HR-MS (ESI) calcd for C_31_H_25_N_2_O_3_ [M + H]^+^ 473.1865, found 473.1869.

*N^3^_,_N^11^-Dimethyl-14-(2-fluorophenyl)-14H-dibenzo[a*,*j]xanthene-3*,*11-dicarboxamide* (**6b**): White solid. Yield 85.4%. m.p. >300 °C. ^1^H-NMR (300 MHz, DMSO-*d*_6_) δ (in ppm): 8.61 (br.q, *J* = 4.3 Hz, 2H, CONH × 2), 8.44 (overlapping d, *J* = 5.5 Hz, 4H), 8.03 (overlapping t, *J* = 7.5 Hz, 4H), 7.58 (overlapping t, *J* = 8.6 Hz, 3H), 7.13–6.92 (m, 3H), 6.86 (s, 1H, H-14), 2.82 (d, *J* = 4.1 Hz, 6H, CH_3_ × 2). ^13^C-NMR (75 MHz, DMSO-*d*_6_) δ (in ppm): 166.8, 159.0 (d, *J* = 244.3 Hz), 149.6, 132.6, 131.9 (d, *J* = 13.3 Hz), 131.4 (d, *J* = 3.2 Hz), 131.0, 131.0, 130.3, 129.5 (d, *J* = 8.3 Hz), 128.8, 125.7, 125.7, 123.0, 118.9, 116.3 (d, *J* = 22.5 Hz), 115.7, 31.6, 26.8. IR (KBr) ν: 3358, 1641, 1622, 1542, 1488, 1465, 1400, 1310, 1253 cm^−1^. HR-MS (ESI) calcd for C_31_H_24_FN_2_O_3_ [M + H]^+^ 491.1771, found 491.1778.

*N^3^_,_N^11^-Dimethyl-14-(4-fluorophenyl)-14H-dibenzo[a*,*j]xanthene-3*,*11-dicarboxamide* (**6c**): White solid. Yield 86.6%. m.p. >300 °C. ^1^H-NMR (300 MHz, DMSO-*d*_6_) δ (in ppm): 8.74 (d, *J* = 8.9 Hz, 2H, H-1, 13), 8.68–8.57 (m, 2H, CONH × 2), 8.45 (br.s, 2H, H-4, 10), 8.03 (overlapping d, *J* = 8.9 Hz, 4H), 7.70–7.58 (m, 4H), 6.97 (t, *J* = 8.7 Hz, 2H, H-3′, 5′), 6.81 (s, 1H, H-14), 2.82 (d, *J* = 4.3 Hz, 6H, CH_3_ × 2). ^13^C-NMR (75 MHz, DMSO-*d*_6_) δ (in ppm): 166.9, 161.0 (d, *J* = 243.3 Hz), 149.3, 142.0, 132.6, 131.1, 130.8, 130.5, 130.2 (d, *J* = 8.0 Hz), 128.7, 125.5, 124.0, 118.9, 117.8, 115.7 (d, *J* = 21.2), 36.0, 26.8. IR (KBr) ν: 3306, 1642, 1549, 1506, 1464, 1401, 1252 cm^−1^. HR-MS (ESI) calcd for C_31_H_24_FN_2_O_3_ [M + H]^+^ 491.1771, found 491.1762.

*N^3^_,_N^11^-Dimethyl-14-(2-chlorophenyl)-14H-dibenzo[a*,*j]xanthene-3*,*11-dicarboxamide* (**6d**): White solid. Yield 89.3%. m.p. >300 °C. ^1^H-NMR (300 MHz, DMSO-*d*_6_) δ (in ppm): 8.60 (overlapping d, *J* = 8.7 Hz, 4H), 8.45 (br.s, 2H, H-4, 10), 8.04 (overlapping d, *J* = 8.8 Hz, 4H), 7.59 (d, *J* = 8.9 Hz, 2H, H-6, 8), 7.49 (d, *J* = 7.3 Hz, 1H, H-6′), 7.32 (d, *J* = 7.7 Hz, 1H, H-3′), 7.11 (t, *J* = 7.4 Hz, 1H, H-5′), 7.03 (t, *J* = 7.2 Hz, 1H, H-4′), 6.79 (s, 1H, H-14), 2.82 (d, *J* = 4.2 Hz, 6H, CH_3_ × 2). ^13^C-NMR (75 MHz, DMSO-*d*_6_) δ (in ppm): 166.8, 149.7, 142.8, 132.7, 132.3, 131.1, 131.0, 130.6, 130.5, 130.4, 129.2, 128.8, 128.7, 125.5, 123.6, 119.0, 116.7, 35.2, 26.8. IR (KBr) ν: 3428, 1640, 1550, 1465, 1400, 1252 cm^−1^. HR-MS (ESI) calcd for C_31_H_24_ClN_2_O_3_ [M + H]^+^ 507.1475, found 507.1481.

*N^3^_,_N^11^-Dimethyl-14-(4-chlorophenyl)-14H-dibenzo[a*,*j]xanthene-3*,*11-dicarboxamide* (**6e**): White solid. Yield 82.7%. m.p. >300 °C. ^1^H-NMR (300 MHz, DMSO-*d*_6_) δ (in ppm): 8.71 (d, *J* = 9.0 Hz, 2H, H-1, 13), 8.56 (br.q, *J* = 4.6 Hz, 2H, CONH × 2), 8.44 (br.s, 2H, H-4, 10), 8.03 (overlapping t, *J* = 8.4 Hz, 4H), 7.67–7.58 (m, 4H), 7.21 (d, *J* = 8.5 Hz, 2H, H-3′, 5′), 6.81 (s, 1H, H-14), 2.83 (d, *J* = 4.5 Hz, 6H, CONCH_3_ × 2). ^13^C-NMR (75 MHz, DMSO-*d*_6_) δ (in ppm): 166.9, 149.4, 144.7, 132.5, 131.6, 131.2, 130.8, 130.5, 130.1, 129.0, 128.6, 125.5, 124.0, 118.9, 117.5, 36.2, 26.8. IR (KBr) ν: 3350, 1642, 1548, 1488, 1465, 1400, 1253 cm^−1^. HR-MS (ESI) calcd for C_31_H_24_ClN_2_O_3_ [M + H]^+^ 507.1475, found 507.1483.

*N^3^_,_N^11^-Dimethyl-14-(3-nitrophenyl)-14H-dibenzo[a*,*j]xanthene-3*,*11-dicarboxamide* (**6f**): White solid. Yield 84.9%. m.p. >300 °C. ^1^H-NMR (300 MHz, DMSO-*d*_6_) δ (in ppm): 8.80 (d, *J* = 9.0 Hz, 2H, H-1, 13), 8.64–8.55 (m, 3H), 8.46 (s, 2H, H-4, 10), 8.10–8.00 (m, 5H), 7.86 (br.d, *J* = 8.0 Hz, 1H, H-4′), 7.67 (d, *J* = 8.9 Hz, 2H, H-6, 8), 7.46 (t, *J* = 8.0 Hz, 1H, H-5′), 7.02 (s, 1H, H-14), 2.82 (d, *J* = 4.4 Hz, 6H, CONCH_3_ × 2). ^13^C-NMR (75 MHz, DMSO-*d*_6_) δ (in ppm): 166.8, 149.5, 148.3, 147.7, 134.8, 132.5, 131.2, 131.2, 130.7, 130.5, 128.8, 125.7, 123.8, 122.5, 122.2, 119.0, 117.0, 36.3, 26.8. IR (KBr) ν: 3449, 3352, 1641, 1546, 1524, 1464, 1401, 1350, 1348, 1255 cm^−1^. HR-MS (ESI) calcd for C_31_H_24_N_3_O_5_ [M + H]^+^ 518.1716, found 518.1722.

*N^3^_,_N^11^-Dimethyl-14-(4-nitrophenyl)-14H-dibenzo[a*,*j]xanthene-3*,*11-dicarboxamide* (**6g**): White solid. Yield 84.2%. m.p. 285–287 °C. ^1^H-NMR (300 MHz, DMSO-*d*_6_) δ (in ppm): 8.75 (d, *J* = 8.9 Hz, 2H, H-1, 13), 8.60 (br.d, *J* = 4.3 Hz, 2H, CONH × 2), 8.45 (s, 2H, H-4, 10), 8.12–7.98 (m, 6H), 7.92 (d, *J* = 8.6 Hz, 2H, H-2′, 6′), 7.65 (d, *J* = 8.9 Hz, 2H, H-6, 8), 6.99 (s, 1H, H-14), 2.82 (d, *J* = 4.0 Hz, 6H, CH_3_ × 2). ^13^C-NMR (75 MHz, DMSO-*d*_6_) δ (in ppm): 166.8, 152.8, 149.4, 146.4, 132.5, 131.2, 131.2, 130.5, 129.5, 128.7, 125.7, 124.3, 123.9, 119.0, 116.7, 36.7, 26.8. IR (KBr) ν: 3353, 1645, 1550, 1523, 1465, 1399, 1344, 1255 cm^−1^. HR-MS (ESI) calcd for C_31_H_24_N_3_O_5_ [M + H]^+^ 518.1716, found 518.1710.

*N^3^_,_N^11^-Dimethyl-14-(4-methylphenyl)-14H-dibenzo[a*,*j]xanthene-3*,*11-dicarboxamide* (**6h**): White solid. Yield 85.7%. m.p. >300 °C.^1^H-NMR (300 MHz, DMSO-*d*_6_) δ (in ppm): 8.71 (d, *J* = 9.0 Hz, 2H, H-1, 13), 8.58 (q, *J* = 4.5 Hz, 2H, CONH × 2), 8.43 (d, *J* = 1.4 Hz, 2H, H-4, 10), 8.04–7.96 (m, 4H), 7.60 (d, *J* = 8.9 Hz, 2H, H-6, 8), 7.48 (d, *J* = 8.1 Hz, 2H, H-2′, 6′), 6.92 (d, *J* = 8.0 Hz, 2H, H-3′, 5′), 6.72 (s, 1H, H-14), 2.82 (d, *J* = 4.5 Hz, 6H, CONCH_3_ × 2), 2.03 (s, 3H, Ar-CH_3_). ^13^C-NMR (75 MHz, DMSO-*d*_6_) δ (in ppm): 166.9, 149.3, 142.9, 136.0, 132.6, 131.0, 130.5, 130.4, 129.5, 128.6, 128.3, 125.3, 124.1, 118.9, 118.0, 36.5, 26.8, 20.9. IR (KBr) ν: 3356, 1641, 1548, 1466, 1400, 1309, 1253 cm^−1^. HR-MS (ESI) calcd for C_32_H_27_N_2_O_3_ [M + H]^+^ 487.2022, found 487.2031.

*N^3^_,_N^11^-Dipropyl-14-phenyl-14H-dibenzo[a*,*j]xanthene-3*,*11-dicarboxamide* (**7a**): White solid. Yield 87.8%. m.p. >300 °C. ^1^H-NMR (400 MHz, DMSO-*d*_6_) δ (in ppm): 8.74 (d, *J* = 8.9 Hz, 2H, H-1, 13), 8.57 (t, *J* = 5.5 Hz, 2H, CONH × 2), 8.44 (s, 2H, H-4, 10), 8.06–8.00 (m, 4H), 7.69–7.55 (m, 4H), 7.15 (t, *J* = 7.6 Hz, 2H, H-3′, 5′), 6.98 (t, *J* = 7.5 Hz, 1H, H-4′), 6.78 (s, 1H, H-14), 3.30–3.13 (m, 4H, NHCH_2_CH_2_CH_3_ × 2), 1.56 (m, 4H, NHCH_2_CH_2_CH_3_ × 2), 0.91 (t, *J* = 7.4 Hz, 6H, NHCH_2_CH_2_CH_3_ × 2). IR (KBr) ν: 3272, 2959, 2927, 1638, 1551, 1463, 1250 cm^−1^. HR-MS (ESI) calcd for C_35_H_33_N_2_O_3_ [M + H]^+^ 529.2491, found 529.2498.

*N^3^_,_N^11^-Dipropyl-14-(2-fluorophenyl)-14H-dibenzo[a*,*j]xanthene-3*,*11-dicarboxamide *(**7b**): White solid. Yield 91.2%. m.p. >300 °C. ^1^H-NMR (400 MHz, DMSO-*d*_6_) δ (in ppm): 8.57 (t, *J* = 5.6 Hz, 2H, CONH × 2), 8.47 (d, *J* = 8.9 Hz, 2H, H-1, 13), 8.46 (s, 2H, H-4, 10), 8.07 (d, *J* = 9.0 Hz, 2H, H-5, 9), 8.04 (dd, *J* = 8.9, 1.5 Hz, 2H, H-2, 12), 7.63 (d, *J* = 8.9 Hz, 2H, H-6, 8), 7.58 (t, *J* = 7.8 Hz, 1H, H-6′), 7.13–7.05 (m, 2H, H-3′, 5′), 7.04–6.90 (m, 1H, H-4′), 6.89 (s, 1H, H-14), 3.34–3.19 (m, 4H, NHCH_2_CH_2_CH_3_ × 2), 1.56 (h, *J* = 7.2 Hz, 4H, NHCH_2_CH_2_CH_3_ × 2), 0.91 (t, *J* = 7.4 Hz, 6H, NHCH_2_CH_2_CH_3_ × 2). IR (KBr) ν: 3270, 2960, 2934, 1632, 1551, 1464, 1252 cm^−1^. HR-MS (ESI) calcd for C_35_H_32_FN_2_O_3_ [M + H]^+^ 547.2397, found 547.2404.

*N^3^_,_N^11^-Dipropyl-14-(4-fluorophenyl)-14H-dibenzo[a*,*j]xanthene-3*,*11-dicarboxamide* (**7c**): White solid. Yield 88.7%. m.p. >300 °C. ^1^H-NMR (400 MHz, DMSO-*d*_6_) δ (in ppm): 8.73 (d, *J* = 9.0 Hz, 2H, H-1, 13), 8.58 (t, *J* = 5.7 Hz, 2H, CONH × 2), 8.45 (d, *J* = 1.6 Hz, 2H, H-4, 10), 8.06–8.00 (m, 4H), 7.76–7.54 (m, 4H), 6.98 (t, *J* = 8.9 Hz, 2H, H-3′, 5′), 6.82 (s, 1H, H-14), 3.35–3.14 (m, 4H, NHCH_2_CH_2_CH_3_ × 2), 1.57 (h, *J* = 7.3 Hz, 4H, NHCH_2_CH_2_CH_3_ × 2), 0.91 (t, *J* = 7.4 Hz, 6H, NHCH_2_CH_2_CH_3_ × 2). IR (KBr) ν: 3292, 2962, 2931, 2873, 1634, 1549, 1507, 1462, 1399, 1248 cm^−1^. HR-MS (ESI) calcd for C_35_H_32_FN_2_O_3_ [M + H]^+^ 547.2397, found 547.2391.

*N^3^_,_N^11^-Dipropyl-14-(2-chlorophenyl)-14H-dibenzo[a*,*j]xanthene-3*,*11-dicarboxamide* (**7d**): White solid. Yield 84.6%. m.p. >300 °C. ^1^H-NMR (400 MHz, DMSO-*d*_6_) δ (in ppm): 8.67 (d, *J* = 9.0 Hz, 2H, H-1, 13), 8.58 (t, *J* = 5.7 Hz, 2H, CONH × 2), 8.47 (d, *J* = 1.5 Hz, 2H, H-4, 10), 8.09 (d, *J* = 9.0 Hz, 2H, H-5, 9), 8.05 (dd, *J* = 8.9, 1.7 Hz, 2H, H-2, 12), 7.65 (d, *J* = 8.9 Hz, 2H, H-6, 8), 7.54 (d, *J* = 7.8 Hz, 1H, H-6′), 7.34 (d, *J* = 6.8 Hz, 1H, H-3′), 7.15 (t, *J* = 7.0 Hz, 1H, H-5′), 7.07 (t, *J* = 7.6 Hz, 1H, H-4′), 6.89 (s, 1H, H-14), 3.35–3.16 (m, 4H, NHCH_2_CH_2_CH_3_ × 2), 1.56 (m, 4H, NHCH_2_CH_2_CH_3_ × 2), 0.91 (t, *J* = 7.4 Hz, 6H, NHCH_2_CH_2_CH_3_ × 2). IR (KBr) ν: 3260, 2960, 2926, 2872, 1639, 1550, 1463, 1398, 1250 cm^−1^. HR-MS (ESI) calcd for C_35_H_32_ClN_2_O_3_ [M + H]^+^ 563.2101, found 563.2112.

*N^3^_,_N^11^-Dipropyl-14-(4-chlorophenyl)-14H-dibenzo[a*,*j]xanthene-3*,*11-dicarboxamide* (**7e**): White solid. Yield 82.5%. m.p. >300 °C. ^1^H-NMR (400 MHz, DMSO-*d*_6_) δ (in ppm): 8.72 (d, *J* = 9.0 Hz, 2H, H-1, 13), 8.58 (t, *J* = 5.7 Hz, 2H, CONH × 2), 8.46 (d, *J* = 1.7 Hz, 2H, H-4, 10), 8.06 (d, *J* = 8.9 Hz, 2H, H-5, 9), 8.03 (dd, *J* = 9.0, 1.8 Hz, 2H, H-2, 12), 7.64 (d, *J* = 8.6 Hz, 2H, H-2′, 6′), 7.63 (d, *J* = 8.9 Hz, 2H, H-6, 8), 7.22 (d, *J* = 8.6 Hz, 2H, H-3′, 5′), 6.82 (s, 1H, H-14), 3.36–3.19 (m, 4H, NHCH_2_CH_2_CH_3_ × 2), 1.57(h, *J* = 7.3 Hz, 4H, NHCH_2_CH_2_CH_3_ × 2), 0.91 (t, *J* = 7.4 Hz, 6H, NHCH_2_CH_2_CH_3_ × 2). IR (KBr) ν: 3302, 2962, 2931, 1636, 1547, 1462, 1399, 1251 cm^−1^. HR-MS (ESI) calcd for C_35_H_32_ClN_2_O_3_ [M + H]^+^ 563.2101, found 563.2096.

*N^3^_,_N^11^-Dipropyl-14-(3-nitrophenyl)-14H-dibenzo[a*,*j]xanthene-3*,*11-dicarboxamide* (**7f**): White solid. Yield 86.3%. m.p. >300 °C. ^1^H-NMR (400 MHz, DMSO-*d*_6_) δ (in ppm): 8.80 (d, *J* = 9.0 Hz, 2H, H-1, 13), 8.62–8.56 (m, 3H), 8.47 (d, *J* = 1.6 Hz, 2H, H-4, 10), 8.17–7.99 (m, 5H), 7.87 (dd, *J* = 8.2, 1.4 Hz, 1H, H-4′), 7.68 (d, *J* = 8.9 Hz, 2H, H-6, 8), 7.47 (t, *J* = 8.0 Hz, 1H, H-5′), 7.03 (s, 1H, H-14), 3.38–3.19 (m, 4H, NHCH_2_CH_2_CH_3_ × 2), 1.56 (m, 4H, NHCH_2_CH_2_CH_3_ × 2), 0.91 (t, *J* = 7.4 Hz, 6H, NHCH_2_CH_2_CH_3_ × 2). IR (KBr) ν: 3243, 2959, 2921, 2868, 1630, 1526, 1462, 1400, 1348, 1319, 1243 cm^−1^. HR-MS (ESI) calcd for C_35_H_32_N_3_O_5_ [M + H]^+^ 574.2342, found 574.2332.

*N^3^_,_N^11^-Dipropyl-14-(4-nitrophenyl)-14H-dibenzo[a*,*j]xanthene-3*,*11-dicarboxamide* (**7g**): White solid. Yield 85.3%. m.p. >300 °C. ^1^H-NMR (400 MHz, DMSO-*d*_6_) δ (in ppm): 8.74 (d, *J* = 9.0 Hz, 2H, H-1, 13), 8.59 (t, *J* = 5.7 Hz, 2H, CONH × 2), 8.46 (d, *J* = 1.7 Hz, 2H, H-4, 10), 8.09 (d, *J* = 9.0 Hz, 2H, H-3′, 5′), 8.06–8.01(m, 4H), 7.93 (d, *J* = 8.9 Hz, 2H, H-2′, 6′), 7.67 (d, *J* = 8.9 Hz, 2H, H-6, 8), 7.00 (s, 1H, H-14), 3.36–3.14(m, 4H, NHCH_2_CH_2_CH_3_ × 2), 1.56 (h, *J* = 7.3 Hz, 4H, NHCH_2_CH_2_CH_3_ × 2), 0.91 (t, *J* = 7.4 Hz, 6H, NHCH_2_CH_2_CH_3_ × 2). IR (KBr) ν: 3273, 2966, 2925, 2872, 1639, 1622, 1529, 1463, 1399, 1345, 1250 cm^−1^. HR-MS (ESI) calcd for C_35_H_32_N_3_O_5_ [M + H]^+^ 574.2342, found 574.2350.

*N^3^_,_N^11^-Dipropyl-14-(4-methylphenyl)-14H-dibenzo[a*,*j]xanthene-3*,*11-dicarboxamide* (**7h**): White solid. Yield 89.9%. m.p. >300 °C. ^1^H-NMR (400 MHz, DMSO-*d*_6_) δ (in ppm): 8.71 (d, *J* = 9.0 Hz, 2H, H-1, 13), 8.57 (t, *J* = 5.7Hz, 2H, CONH × 2), 8.44 (d, *J* = 1.6 Hz, 2H, H-4, 10), 8.03 (d, *J* = 8.9 Hz, 2H, H-5, 9), 8.01 (dd, *J* = 9.0, 1.8 Hz, 2H, H-2, 12), 7.62 (d, *J* = 8.9 Hz, 2H, H-6, 8), 7.49 (d, *J* = 8.1 Hz, 2H, H-2′, 6′), 6.94 (d, *J* = 8.0 Hz, 2H, H-3′, 5′), 6.73 (s, 1H, H-14), 3.33–3.16 (m, 4H, NHCH_2_CH_2_CH_3_ × 2), 2.05 (s, 3H, Ar-CH_3_), 1.57 (h, *J* = 7.3 Hz, 4H, NHCH_2_CH_2_CH_3_ × 2), 0.91 (t, *J* = 7.4 Hz, 6H, NHCH_2_CH_2_CH_3_ × 2). IR (KBr) ν: 3289, 2959, 2925, 2856, 1639, 1552, 1511, 1462, 1398, 1317, 1241 cm^−1^. HR-MS (ESI) calcd for C_36_H_35_N_2_O_3_ [M + H]^+^ 543.2648, found 543.2640.

*N^3^_,_N^11^-Diisobutyl-14-phenyl-14H-dibenzo[a*,*j]xanthene-3*,*11-dicarboxamide* (**8a**): White solid. Yield 84.3%. m.p. >300 °C. ^1^H-NMR (400 MHz, DMSO-*d*_6_) δ (in ppm): 8.74 (d, *J* = 9.0 Hz, 2H, H-1, 13), 8.58 (t, *J* = 5.8 Hz, 2H, CONH × 2), 8.45 (d, *J* = 1.1 Hz, 2H, H-4, 10), 8.07–8.00 (m, 4H), 7.64 (d, *J* = 8.9 Hz, 2H, H-6, 8), 7.63 (d, *J* = 7.5 Hz, 2H, H-2′, 6′), 7.15 (t, *J* = 7.7 Hz, 2H, H-3′, 5′), 6.98 (t, *J* = 7.3 Hz, 1H, H-4′), 6.78 (s, 1H, H-14), 3.22–3.03 (m, 4H, NHCH_2_ × 2), 1.94–1.82 (m, 2H, CH(CH_3_)_2_ × 2), 0.91 (d, *J* = 6.7 Hz, 12H, CH(CH_3_)_2_ × 2). IR (KBr) ν: 3298, 2958, 2924, 1635, 1550, 1462, 1242 cm^−1^. HR-MS (ESI) calcd for C_37_H_37_N_2_O_3_ [M + H]^+^ 557.2804, found 557.2809.

*N^3^_,_N^11^-Diisobutyl-14-(2-fluorophenyl)-14H-dibenzo[a*,*j]xanthene-3*,*11-dicarboxamide* (**8b**): White solid. Yield 82.7%. m.p. >300 °C. ^1^H-NMR (400 MHz, DMSO-*d*_6_) δ (in ppm): 8.57 (t, *J* = 5.8 Hz, 2H, CONH × 2), 8.50–8.44 (m, 4H), 8.08 (d, *J* = 9.0 Hz, 2H, H-5, 9), 8.05 (dd, *J* = 9.0, 1.6 Hz, 2H, H-2, 12), 7.63 (d, *J* = 8.9 Hz, 2H, H-6, 8), 7.58 (t, *J* = 7.8 Hz, 1H, H-6′), 7.17–7.05 (m, 2H, H-3′, 5′), 7.04–6.95 (m, 1H, H-4′), 6.89 (s, 1H, H-14), 3.17–3.09 (m, 4H, NHCH_2_ × 2), 1.94–1.81 (m, 2H, CH(CH_3_)_2_ × 2), 0.91 (d, *J* = 6.7 Hz, 12H, CH(CH_3_)_2_ × 2). IR (KBr) ν: 3295, 2958, 2925, 1637, 1550, 1463, 1251 cm^−1^. HR-MS (ESI) calcd for C_37_H_36_FN_2_O_3_ [M + H]^+^ 575.2710, found 575.2715.

*N^3^_,_N^11^-Diisobutyl-14-(4-fluorophenyl)-14H-dibenzo[a*,*j]xanthene-3*,*11-dicarboxamide* (**8c**): White solid. Yield 83.8%. m.p. >300 °C. ^1^H-NMR (400 MHz, DMSO-*d*_6_) δ (in ppm): 8.73 (d, *J* = 9.0 Hz, 2H, H-1, 13), 8.59 (t, *J* = 5.8 Hz, 2H, CONH × 2), 8.46 (d, *J* = 1.6 Hz, 2H, H-4, 10), 8.07–8.00 (m, 4H), 7.76–7.54 (m, 4H), 6.98 (t, *J* = 8.9 Hz, 2H, H-3′, 5′), 6.82 (s, 1H, H-14), 3.21–3.07 (m, 4H, NHCH_2_ × 2), 1.94–1.81 (m, 2H, CH(CH_3_)_2_ × 2), 0.91 (d, *J* = 6.7 Hz, 12H, CH(CH_3_)_2_ × 2). IR (KBr) ν: 3299, 2958, 2925, 1635, 1549, 1507, 1462, 1399, 1248 cm^−1^. HR-MS (ESI) calcd for C_37_H_36_FN_2_O_3_ [M + H]^+^ 575.2710, found 575.2719.

*N^3^_,_N^11^-Diisobutyl-14-(2-chlorophenyl)-14H-dibenzo[a*,*j]xanthene-3*,*11-dicarboxamide* (**8d**): White solid. Yield 87.5%. m.p. >300 °C. ^1^H-NMR (400 MHz, DMSO-*d*_6_) δ (in ppm): 8.66 (d, *J* = 9.0 Hz, 2H, H-1, 13), 8.59 (t, *J* = 5.8 Hz, 2H, CONH × 2), 8.47 (d, *J* = 1.3 Hz, 2H, H-4, 10), 8.12–8.03 (m, 4H), 7.64 (d, *J* = 8.9 Hz, 2H, H-6, 8), 7.53 (d, *J* = 7.7 Hz, 1H, H-6′), 7.34 (d, *J* = 8.0 Hz, 1H, H-3′), 7.14 (t, *J* = 7.1 Hz, 1H, H-5′), 7.07 (t, *J* = 6.9 Hz, 1H, H-4′), 6.87 (s, 1H, H-14), 3.13 (t, *J* = 6.4 Hz, 4H, NHCH_2_ × 2), 1.94–1.81 (m, 2H, CH(CH_3_)_2_ × 2), 0.91 (d, *J* = 6.7 Hz, 12H, CH(CH_3_)_2_ × 2). IR (KBr) ν: 3281, 2960, 2926, 1638, 1547, 1463, 1397, 1249 cm^−1^. HR-MS (ESI) calcd for C_37_H_36_ClN_2_O_3_ [M + H]^+^ 591.2414, found 591.2420.

*N^3^_,_N^11^-Diisobutyl-14-(4-chlorophenyl)-14H-dibenzo[a*,*j]xanthene-3*,*11-dicarboxamide* (**8e**): White solid. Yield 81.6%. m.p. 266–268 °C. ^1^H-NMR (400 MHz, DMSO-*d*_6_) δ (in ppm): 8.72 (d, *J* = 9.0 Hz, 2H, H-1, 13), 8.58 (t, *J* = 5.8 Hz, 2H, CONH × 2), 8.46 (d, *J* = 1.7 Hz, 2H, H-4, 10), 8.06 (d, *J* = 8.9 Hz, 2H, H-5, 9), 8.03 (dd, *J* = 8.9, 1.8 Hz, 2H, H-2, 12), 7.64 (d, *J* = 8.6 Hz, 2H, H-2′, 6′), 7.63 (d, *J* = 8.9 Hz, 2H, H-6, 8), 7.22 (d, *J* = 8.6 Hz, 2H, H-3′, 5′), 6.82 (s, 1H, H-14), 3.20–3.06 (m, 4H, NHCH_2_ × 2), 1.93–1.80 (m, 2H, CH(CH_3_)_2_ × 2), 0.91 (d, *J* = 6.7 Hz, 12H, CH(CH_3_)_2_ × 2). IR (KBr) ν: 3319, 2958, 2926, 1637, 1546, 1463, 1398, 1250 cm^−1^. HR-MS (ESI) calcd for C_37_H_36_ClN_2_O_3_ [M + H]^+^ 591.2414, found 591.2418.

*N^3^_,_N^11^-Diisobutyl-14-(3-nitrophenyl)-14H-dibenzo[a*,*j]xanthene-3*,*11-dicarboxamide* (**8f**): White solid. Yield 85.1%. m.p. >300 °C. ^1^H-NMR (400 MHz, DMSO-*d*_6_) δ (in ppm): 8.80 (d, *J* = 9.0 Hz, 2H, H-1, 13), 8.62–8.56 (m, 3H), 8.47 (d, *J* = 1.6 Hz, 2H, H-4, 10), 8.17–7.99 (m, 5H), 7.87 (dd, *J* = 8.2, 1.4 Hz, 1H, H-4′), 7.68 (d, *J* = 8.9 Hz, 2H, H-6, 8), 7.48 (t, *J* = 8.0 Hz, 1H, H-5′), 7.03 (s, 1H, H-14), 3.23–3.02 (m, 4H, NHCH_2_ × 2), 1.94–1.81 (m, 2H, CH(CH_3_)_2_ × 2), 0.91 (d, *J* = 6.7 Hz, 12H, CH(CH_3_)_2_ × 2). IR (KBr) ν: 3293, 2960, 2926, 1640, 1532, 1462, 1396, 1350, 1246 cm^−1^. HR-MS (ESI) calcd for C_37_H_36_N_3_O_5_ [M + H]^+^ 602.2655, found 602.2650.

*N^3^_,_N^11^-Diisobutyl-14-(4-nitrophenyl)-14H-dibenzo[a*,*j]xanthene-3*,*11-dicarboxamide* (**8g**): White solid. Yield 82.6%. m.p. >300 °C. ^1^H-NMR (400 MHz, DMSO-*d*_6_) δ (in ppm): 8.74 (d, *J* = 8.9 Hz, 2H, H-1, 13), 8.59 (t, *J* = 5.7 Hz, 2H, CONH × 2), 8.47 (d, *J* = 1.6 Hz, 2H, H-4, 10), 8.09 (d, *J* = 9.0 Hz, 2H, H-3′, 5′), 8.06–8.01(m, 4H), 7.93 (d, *J* = 8.9 Hz, 2H, H-2′, 6′), 7.67 (d, *J* = 8.9 Hz, 2H, H-6, 8), 7.00 (s, 1H, H-14), 3.23–3.02 (m, 4H, NHCH_2_ × 2), 1.94–1.81 (m, 2H, CH(CH_3_)_2_ × 2), 0.91 (d, *J* = 6.7 Hz, 12H, CH(CH_3_)_2_ × 2). IR (KBr) ν: 3320, 2958, 2926, 1640, 1546, 1514, 1463, 1346, 1250 cm^−1^. HR-MS (ESI) calcd for C_37_H_36_N_3_O_5_ [M + H]^+^ 602.2655, found 602.2648.

*N^3^_,_N^11^-Diisobutyl-14-(4-methylphenyl)-14H-dibenzo[a*,*j]xanthene-3*,*11-dicarboxamide* (**8h**): White solid. Yield 84.6%. m.p. >300 °C. ^1^H-NMR (400 MHz, DMSO-d6) δ (in ppm): 8.71 (d, *J* = 9.0 Hz, 2H, H-1, 13), 8.57 (t, *J* = 5.8 Hz, 2H, CONH × 2), 8.45 (d, *J* = 1.6 Hz, 2H, H-4, 10), 8.03 (d, *J* = 8.9 Hz, 2H, H-5, 9), 8.01 (dd, *J* = 8.9, 1.8 Hz, 2H, H-2, 12), 7.62 (d, *J* = 8.9 Hz, 2H, H-6, 8), 7.49 (d, *J* = 8.1 Hz, 2H, H-2′, 6′), 6.94 (d, *J* = 8.0 Hz, 2H, H-3′, 5′), 6.73 (s, 1H, H-14), 3.23–3.02 (m, 4H, NHCH_2_ × 2), 2.05 (s, 3H, Ar-CH_3_), 1.94–1.81 (m, 2H, CH(CH_3_)_2_ × 2), 0.91 (d, *J* = 6.7 Hz, 12H, CH(CH_3_)_2_ × 2). IR (KBr) ν: 3340, 2956, 2922, 1641, 1548, 1463, 1397, 1316, 1249 cm^−1^. HR-MS (ESI) calcd for C_38_H_39_N_2_O_3_ [M + H]^+^ 571.2961, found 571.2967.

The ^1^H-NMR for compound **5a**–**8h** and ^13^C-NMR for compound **6a**–**h** can be found in Supplementary Materials.

### 2.2. Cytotoxicity Assay

The synthesized compounds **5a**–**h**, **6a**–**h**, **7a**–**h** and **8a**–**h** were tested for cytotoxicity in four human cancer cell lines, which contained human hepatoma cells (SK-HEP-1, HepG2, SMMC-7721) and acute promyelocytic leukemia cells (NB4), and arsenic trioxide (As_2_O_3_) was used as positive control. In addition, the dibenzo[*a*,*j*]xanthene derivatives **9a**–**d** (the preparation method of compounds **9a**–**d** according [15], Figure 2), which do not possess functional groups on the 3- and 11-positions, were used as negative standards. The IC_50_ values of the carboxamide derivatives for antiproliferative activity are listed in Table 1.

## 3. Discussion

### 3.1. Synthesis of Target Compounds

Compound **3** was mixed with Br_2_ in acetic acid at room temperature, and then the mixture was refluxed for 3 h during which period three portions of Sn were added; compound 6-bromo-2-naphthol (yield 75%) was obtained by substitution and reduction reactions of compound **3** [12,13]. The mixture of 6-bromo-2-naphthol and CuCN in DMF was heated at 160–170 °C for 4 h under nitrogen atmosphere to give the compound 6-cyano-2-naphthol (yield 83%) [14]. The latter was refluxed for 8 h in a solvent of 10% hydrochloric acid, the newly formed precipitate was filtered and washed with water and EtOAc successively, and then dried to achieve the compound 6-hydroxy-2-naphthalenecarboxylic acid (yield 75%) [12]. A mixture of the naphthalenecarboxylic acid (20 mmol), and an appropriate amount of arylaldehyde (10.5 mmol), glacial acetic acid (20 mL) and concentrated sulfuric acid (1 mL) was stirred at room temperature for 10 min, and then refluxed for 0.5–2 h to afford compounds **4a**–**h** (yield 82–88%) [12].

Compounds **4a**–**h** were firstly converted into the corresponding acyl chlorides (intermediates), and then the intermediates were converted into corresponding dicarboxamides **5a**–**h** with excessive gaseous NH_3_; they are easy to purify because they have little solubility in CHCl_3_ which was used as the solvent, and the reactants are soluble. The acyl chlorides were converted into corresponding dicarboxamides **6a**–**h** in the presence of excessive methylamine in aqueous solution. The principle for the purification of compounds **6a**–**h** is the same as that for compounds **5a**–**h**. Target compounds **7a**–**h** were achieved via the reaction of the acyl chlorides with propylamine at room temperature for 2–3 h, and the purification process was also the same as that for compounds **5a**–**h**, but with less solvent CHCl_3_, in order to improve the yield of the product. This is because the solubility of **7a**–**h** in chloroform is larger than that of **5a**–**h** in chloroform, and the increased liposolubility is due to the introduction of the propyl group to the N atom in the molecule. Variation of the above solubility is better reflected by compounds **8a**–**h** with an isobutyl group on the N atom in the molecule, which can be completely dissolved in the reaction mixture. The processing of compounds **8a**–**h** was completely different from that of the previous samples. The reaction solution was washed with brine and distilled water successively, and then the organic layer was dried with MgSO_4_, filtered and evaporated to give the crude product, which was recrystallized in petroleum ether-EtOAc to obtain compounds **8a**–**h** (Scheme 1).

The structures of synthesized compounds **5a**–**h**, **6a**–**h**, **7a**–**h** and **8a**–**h** were confirmed by ^1^H-NMR, HRMS and IR spectra, and **6a**–**h** were further confirmed by ^13^C-NMR spectra. Since compounds **5a**–**8h** have symmetric structures, the chemical shifts of H-1 and H-13, H-2 and H-12, H-4 and H-10, H-5 and H-9, H-6 and H-8 are identical in the ^1^H-NMR spectra, respectively. The H-1, H-2 and H-4 intercouple with each other, and the splitting of the peaks in the spectra and the coupling constants are different. H-1 (d, *J* ≈ 9Hz), H-2 (dd, *J*_1_ ≈ 9Hz, *J*_2_ < 2 Hz), and H-4 (d, *J* < 2 Hz) can be distinguished and assigned. The assignment of the H-5 and H-6 is easy to carry out. H-5 and H-6 are in the meta and ortho positions of the oxygen atom (O-7), respectively, and due to the electron-donating effect of the oxygen atom, the chemical shift of H-5 is greater than that of H-6. The hydrogen atoms on the 14-phenyl group are easy to distinguish. In most cases, the chemical shifts of the hydrogen atoms on the 14-phenyl group have less value than those of hydrogen atoms on naphthalene rings, and the chemical shifts of H-2' and H-6' are greater than those of H-3′ and H-5′, without the influence of other substituents. Although H-14 is hydrogen bonded to a saturated carbon atom, which is at the α-position of three phenyl rings, its chemical shift is relatively large (δ_H-14_ > 6.7).

Since the aromatic carboxamide groups of compounds **5a**–**h** cannot rotate freely, in the ^1^H-NMR spectroscopy, the two hydrogen atoms on the same nitrogen atom exhibit two single peaks with different chemical shifts. The two carboxamide groups of compounds **5a**–**h** are symmetric, so the integral value of each single peak was 2.

### 3.2. Cytotoxicity Assay

As evidenced by the cytotoxicity data in Table 1, compounds **5a**–**h**, **6a**–**h**, **7a**–**h** and **8a**–**h** show better inhibitory activity than **9a**–**d**, and the latter do not show significant antitumor activity (IC_50_ > 50 μM). In general, compounds **5a**–**h** and **6a**–**h** had better antitumor activity than **7a**–**h** and **8a**–**h,** especially the derivatives **6c**–**6e**. The latter proved to be the most potent cytotoxic agents to NB4 cancer cells, the IC_50_ values of which were 0.52 μM and 0.76 μM, respectively, much lower than 5.31 μM of As_2_O_3_. In addition, most of the target compounds exhibited more inhibitory activity on the NB4 cancer cell line than on the other cell lines.

The different antitumor activities of the target molecules to four kinds of tumor cell lines may be attributed to multiple factors such as the side chains on the C-3 and C-11 positions, the nature of the substituent at the 14-phenyl group, the nature of the tested cell lines, and so on.

The carboxamide side chains on C-3 and C-11 exert a strong effect on the antitumor activity. It is found that a small substituent on the N atom of the carboxamide side chain is favorable for cytotoxicity, and the order of inhibitory activity against tumor cells is CH_3_ > (CH_2_)_2_CH_3_ > CH_2_CH(CH_3_)_2_. When a small substituent connects to the N atom, the latter is easier to form a hydrogen bond with the acceptor (**6a** vs. **7a** and **8a**, **6c** vs. **7c** and **8c**, **6e** vs. **7e** and **8e**, Table 1).

The antitumor activities of compounds **5a**–**h** and **6a**–**h** cannot be compared simply in terms of strength or weakness (for example, compound** 5a** shows better inhibitory activity than **6a** to the four cell lines, but compound **6c** exhibits stronger inhibitory activity than **5c** to the HepG2, SMMC-7721 and NB4 cell lines), which may be due to the small size difference between the hydrogen atom and the methyl group on the N atom, so that it does not affect the formation of hydrogen bonds between the amide group and antitumor cells.

A substituent at C-4′ (*para*) position on the 14-phenyl group of the dicarboxamide derivatives showed better inhibitory activity than the other positions (C-2′ and C-3′) in most cases (compounds **5c**–**8c** vs. compounds **5b**–**8b**, compounds **5e**–**8e** vs. compounds **5d**–**8d**, and compounds **5g**–**8g** vs. compounds **5f**–**8f**, Table 1), which may be because the C-4′ substituent at one end of the molecule can combine with tumor cells more easily. A halogen atom was more potent than other substituents at the C-4′ position of the 14-phenyl ring, which may be due to its smaller size as well as its higher capability of forming hydrogen bonds with the tumor cells.

Several compounds show an obvious inhibition selectivity to tumor cells. For example, compound **6d** exhibits anti-proliferative activity toward HepG2 and NB4 cells; however, it does not show cytotoxic activity toward human hepatoma SK-HEP-1 cells (the IC_50_ value was greater than 50 μM, Table 1). Similar cell selectivity can be found for other compounds such as **5d**–**6a**.

## 4. Materials and Methods

### 4.1. General

The melting points of the targeted compounds were determined with an X-6 melting point apparatus (Beijing Tektronix Instrument Co., Ltd., Beijing, China) and were uncorrected. FT-IR spectra were recorded on an Avatar 370 FT-IR spectrometer in the form of KBr pellets (Thermo Nicolet Corporation, Madison, WI, USA). ^1^H-NMR (300 MHz or 400 MHz) and ^13^C-NMR (75 MHz) spectra were recorded on a Bruker Avance 300 (or 400) spectrometer (Bruker Company, Billerica, MA, United States) in DMSO-*d*_6_ solution, using tetramethylsilane (TMS) as an internal standard. HR-MS were measured on a Waters LCT Premier XE benchtop orthogonal acceleration time-of-flight mass spectrometer (Waters Corporation, Milford, MA, USA). Unless otherwise noted, all common solvent and chemicals were purchased from commercial suppliers and used without further purification.

### 4.2. General Procedure for the Preparation of 14-Aryl-14H-dibenzo[a,j]xanthene-3,11-dicarboxamide (***5a***–***h***)

The 4 mmol compounds **4**, 30 mL of CHCl_3_ and 1 drop of dimethylformamide were mixed, and then a solution of 4.4 mL (60 mmol) thionyl chloride in CHCl_3_ (10 mL) was added to the above system, and the mixture was heated to reflux for 4 h. After the solvent and excessive thionyl chloride were removed using a rotary evaporator, and the acyl chlorides can be obtained. The acyl chloride was dissolved in CHCl_3_ (30 mL) at room temperature, then excessive gaseous NH_3_ was introduced into the above solution. The mixture was stirred at room temperature for 2–3 h. A white solid formed was filtered, washed with CHCl_3_ and water successively, and then dried under vacuum to achieve compounds **5a**–**h**.

### 4.3. General Procedure for the Preparation of N^3^,N^11^-Dimethyl-14-aryl-14H-dibenzo[a,j]xanthene-3,11-dicarboxamide (***6a***–***h***)

The acyl chloride (prepared from 4 mmol compound **4** according to the above method) was dissolved in CHCl_3_ (30 mL), and the solution was dropwise added into 10 mL of a 40% methylamine solution in water. The reaction mixture was then stirred at room temperature, and thin-layer chromatography (TLC) was used to monitor the reactions. After stirring for 2–3 h, a white solid formed was filtered, washed with CHCl_3_ and water, separately. After vacuum drying, pure compounds **6a**–**h** were obtained.

### 4.4. General Procedure for the Preparation of N^3^,N^11^-Dipropyl-14-aryl-14H-dibenzo[a,j]xanthene-3,11-dicarboxamide (***7a***–***h***)

The acyl chloride (prepared from 4 mmol compound **4** according to the above method) was dissolved in CHCl_3_ (20 mL), and the solution was dropwise added into a mixed solution of 10 mL CHCl_3 _and 16 mmol propylamine. The reaction mixture was stirred at room temperature for 2–3 h, and TLC was used to monitor the reactions. The white solid formed was filtered, washed with CHCl_3_ and water, separately. The solid was recrystallized from petroleum ether–EtOAc (3:1) to give compounds **7a**–**h**.

### 4.5. General Procedure for the Preparation of N^3^,N^11^-Diisobutyl-14-aryl-14H-dibenzo[a,j]xanthene-3,11-dicarboxamide (***8a***–***h***)

The acyl chloride (prepared from 4 mmol compound **4** according to the above method) was dissolved in CHCl_3_ (30 mL), and the solution was dropwise added into a mixed solution of 20 mL CHCl_3 _and 16 mmol isobutylamine. The reaction mixture was stirred at room temperature, and TLC was used to monitor the reaction. Finally the reaction mixture was washed with brine and water successively, and then dried (MgSO_4_), filtered and concentrated to give the crude products, which were recrystallized from petroleum Ether–EtOAc (6:1) to afford compounds **8a**–**h**.

## 5. Conclusions

In this paper, 32 *N*-substituted14-aryl-14*H*-dibenzo[*a*,*j*]xanthene-3,11-dicarboxamide derivatives were designed and synthesized in acceptable overall yields. All compounds were screened for their cytotoxicity against HepG2, SK-HEP-1, SMMC-7721 and NB4 cell lines. The results of the antitumor activity experiments revealed that some of the compounds exhibit promising inhibitory activity, among which compounds **6c**–**6e** are 10-fold and seven-fold more active compared with the positive control As_2_O_3_ against NB4 cells, respectively. In summary, the SARs show that unsubstituted and methyl-substituted dicarboxamide derivatives on the N atoms of carboxamide side chains are favorable for cytotoxicity, and the introduction of bigger substitutes including *n*-propyl and isobutyl groups leads to inferior results. Based on our previous results [7] and the results in this paper, it is concluded that introducing small-sized groups or polar groups to the N atoms of carboxamide side chains in the dicarboxamide derivatives may be beneficial to antitumor activity.

## Figures and Tables

**Table 1:** IC_50_ values (μM) of the synthesized compounds for antiproliferative activity.

Compound	IC_50_ (μM) for Different Cell Lines ^a^			
	HepG2	SK-HEP-1	SMMC-7721	NB4
**5a**	17.97 ± 0.51	20.86 ± 1.12	9.05 ± 0.23	9.47 ± 0.35
**5b**	>50	24.06 ± 2.06	20.5 ± 1.77	>50
**5c**	9.68 ± 0.35	9.73 ± 0.53	11.76 ± 0.87	8.25 ± 0.34
**5d**	>50	26.24 ± 2.87	10.47 ± 0.76	>50
**5e**	11.58 ± 1.05	9.82 ± 0.19	8.01 ± 0.39	8.12 ± 0.23
**5f**	>50	40.32 ± 1.77	29.49 ± 2.50	>50
**5g**	>50	32.85 ± 3.13	>50	>50
**5h**	36.16 ± 3.91	17.19 ± 0.89	9.84 ± 0.43	8.73 ± 0.53
**6a**	20.31 ± 2.09	>50	14.52 ± 1.83	10.32 ± 1.02
**6b**	30.56 ± 2.98	>50	21.23 ± 2.41	14.54 ± 1.98
**6c**	6.12 ± 0.25	12.23 ± 1.26	7.32 ± 0.49	0.52 ± 0.032
**6d**	9.21 ± 0.88	>50	40.5 ± 2.96	11.6 ± 1.78
**6e**	6.32 ± 0.30	14.61 ± 0.96	8.15 ± 0.46	0.76 ± 0.041
**6f**	40.14 ± 3.21	>50	22.15 ± 1.46	23.34 ± 1.57
**6g**	34.22 ± 3.11	>50	14.63 ± 2.83	9.12 ± 0.37
**6h**	8.76 ± 0.47	20.17 ± 1.19	9.54 ± 0.31	1.63 ± 0.041
**7a**	30.21 ± 2.88	>50	18.86 ± 1.70	23.32 ± 2.42
**7b**	35.67 ± 2.09	>50	41.31 ± 2.11	29.76 ± 2.80
**7c**	9.54 ± 0.31	12.78 ± 0.14	7.43 ± 0.36	7.88 ± 0.16
**7d**	11.79 ± 0.23	>50	>50	12.61 ± 0.82
**7e**	8.95 ± 0.31	15.67 ± 0.15	12.20 ± 0.89	13.83 ± 0.37
**7f**	>50	>50	34.12 ± 1.55	30.88 ± 1.80
**7g**	22.13 ± 1.30	>50	19.73 ± 0.78	10.97 ± 0.45
**7h**	9.08 ± 0.21	22.56 ± 0.45	16.87 ± 1.38	8.87 ± 0.35
**8a**	32.34 ± 1.25	>50	19.76 ± 0.83	27.78 ± 2.61
**8b**	40.45 ± 2.52	>50	>50	32.48 ± 1.01
**8c**	10.50 ± 0.35	13.56 ± 0.23	15.06 ± 0.88	9.85 ± 0.58
**8d**	12.72 ± 0.91	>50	>50	13.76 ± 1.17
**8e**	11.34 ± 0.50	17.75 ± 0.35	13.30 ± 0.86	15.34 ± 1.56
**8f**	>50	>50	>50	32.65 ± 2.21
**8g**	25.65 ± 1.92	>50	21.80 ± 1.53	12.83 ± 0.59
**8h**	10.37 ± 0.49	23.67 ± 0.34	17.89 ± 1.33	10.01 ± 0.41
**9a–d**	> 50	> 50	> 50	> 50
**As_2_O_3_**	5.92 ± 0.21	6.23 ± 0.32	9.43 ± 0.50	5.31 ± 0.22

^a^ Values are means ± standard deviation from three independent experiments.

## Supplementary Materials

The following are available online at www.mdpi.com/1420-3049/22/4/517/s1.
