# Supplementary material for: Synthesis and Cytotoxicity of N-Substituted Dibenzo[a,j]xanthene-3,11-dicarboxamide Derivatives
Source: Molecules. 2017 Mar 23;22(4):517. doi: 10.3390/molecules22040517 (PMC6154592; doi:10.3390/molecules22040517)
Supplement: Supplementary file 1 [file molecules-22-00517-s001.pdf]

Synthesis and Cytotoxicity of *N*-Substituted Dibenzo[*a,j*]xanthene-3,11-dicarboxamide Derivatives

Yongbin Song, Yihui Yang, Lijun Wu, Naiwei Dong, Shang Gao, Hongrui Ji, Xia Du, Bo Liu and Guoyou Chen

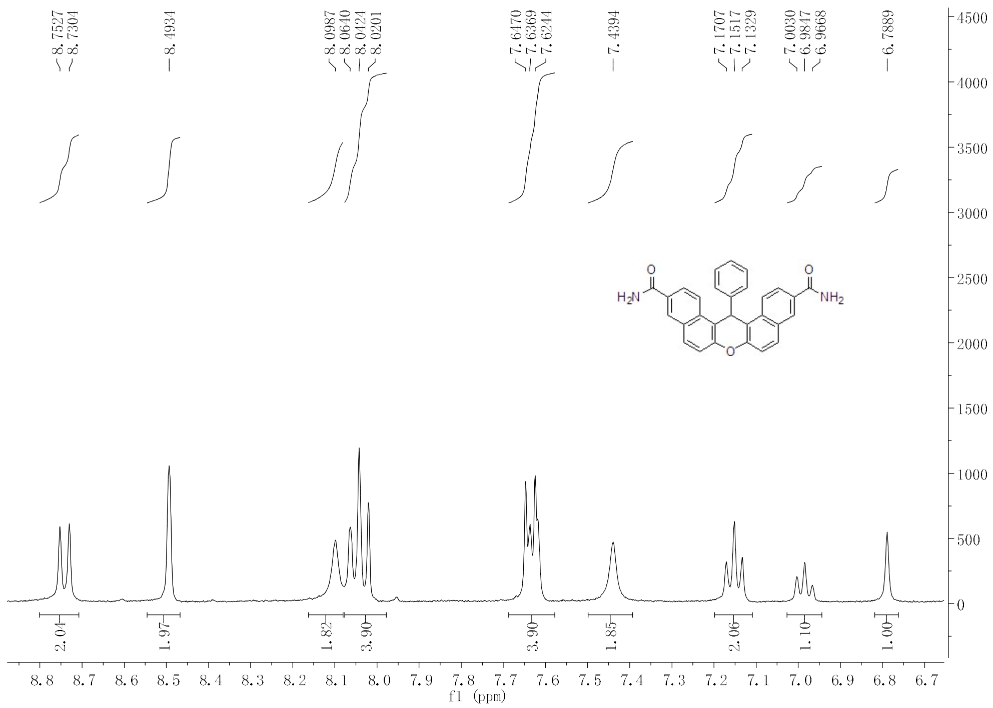

^{1}H-NMR spectrum of **5a**

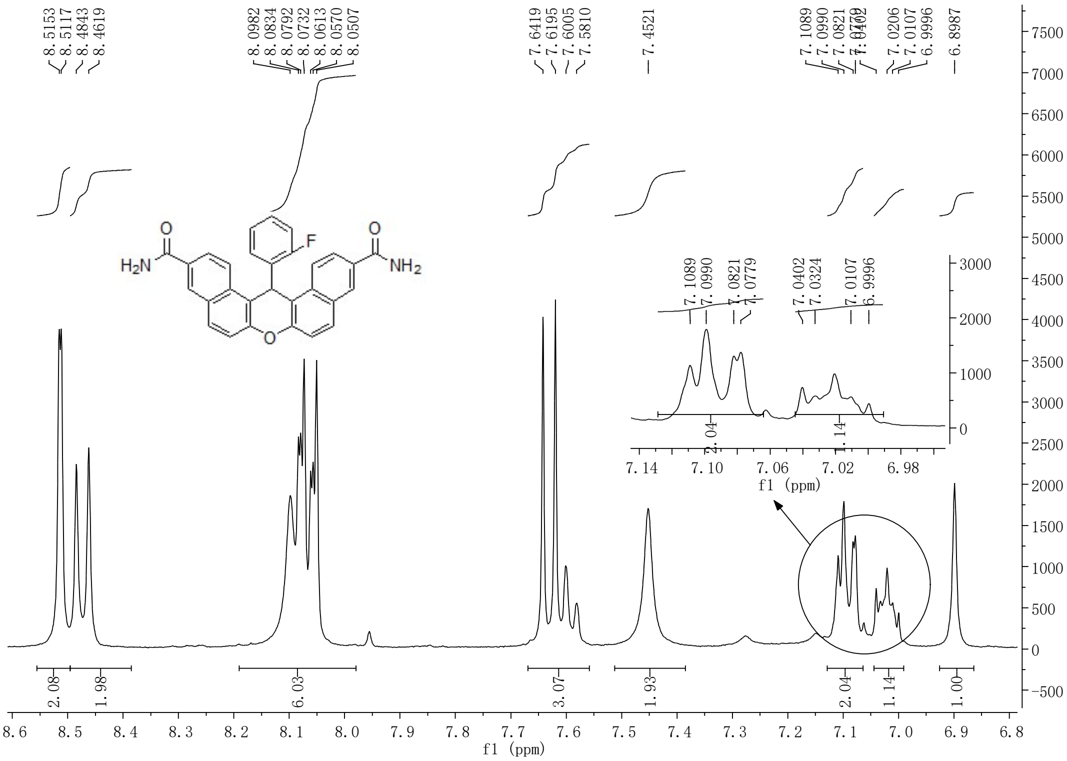

^{1}H-NMR spectrum of **5b**

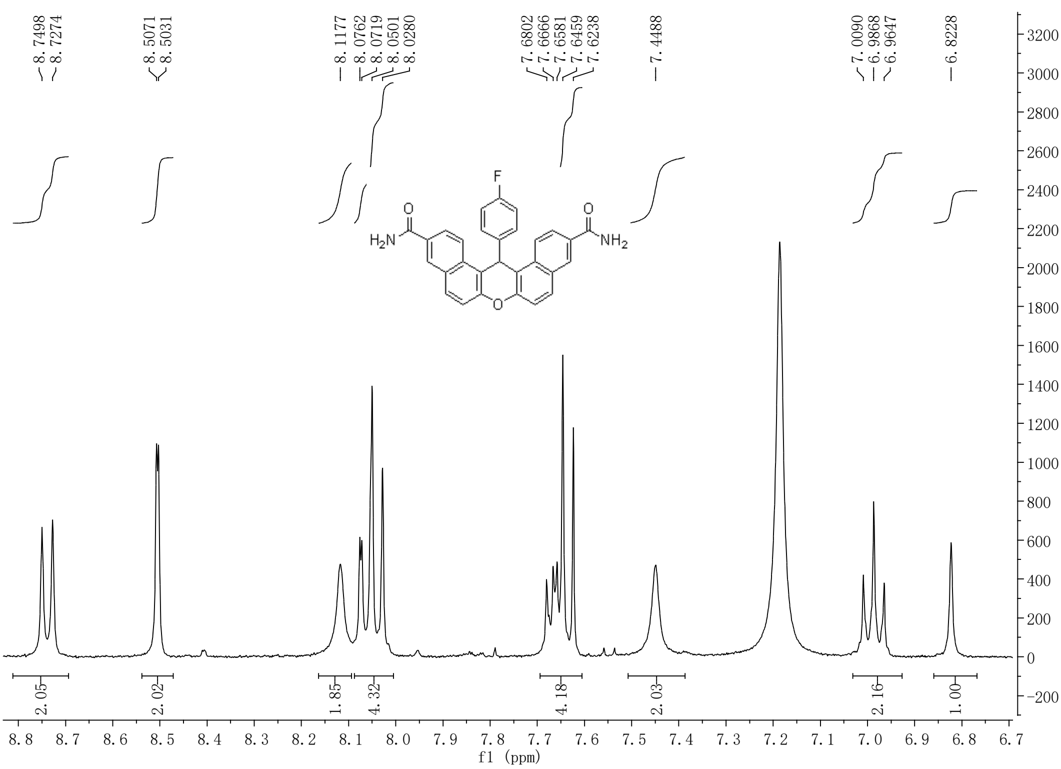

^{1}H-NMR spectrum of **5c**

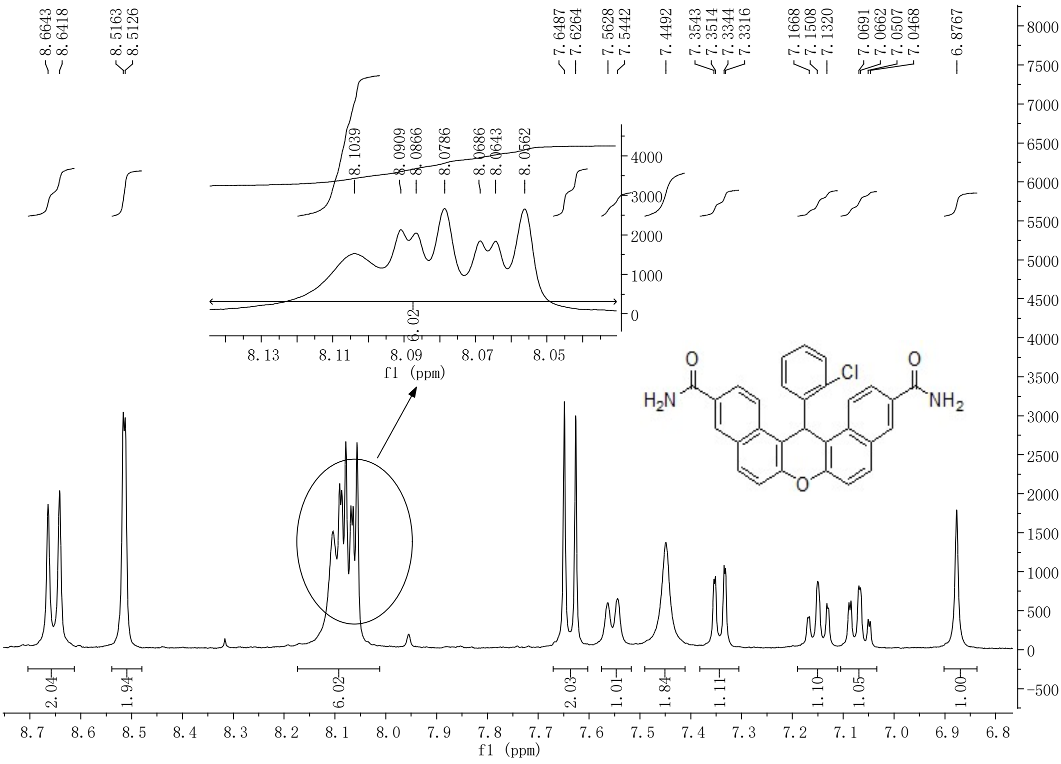

^{1}H-NMR spectrum of **5d**

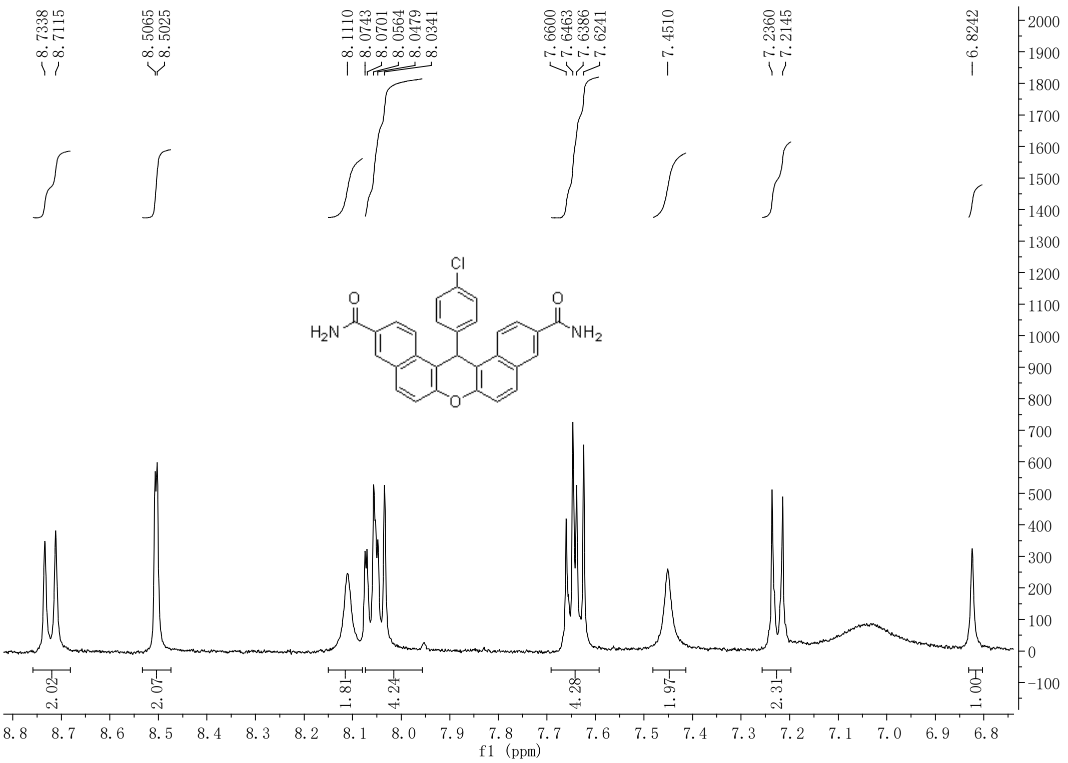

^{1}H-NMR spectrum of **5e**

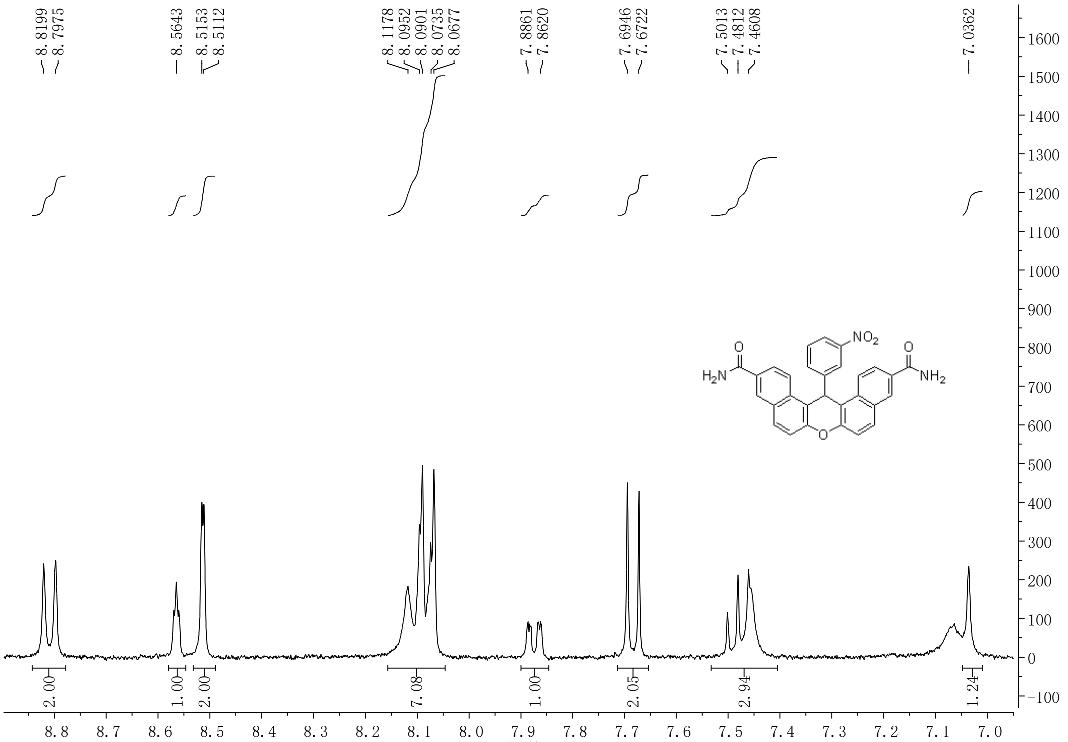

^{1}H-NMR spectrum of **5f**

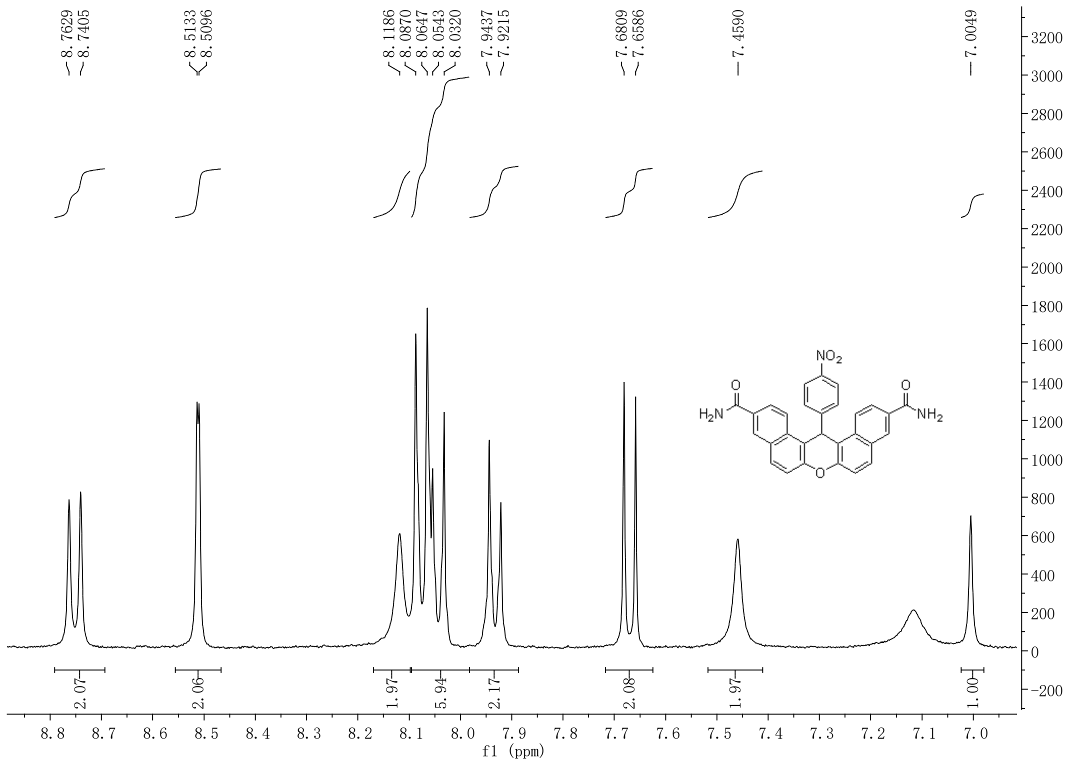

^{1}H-NMR spectrum of **5g**

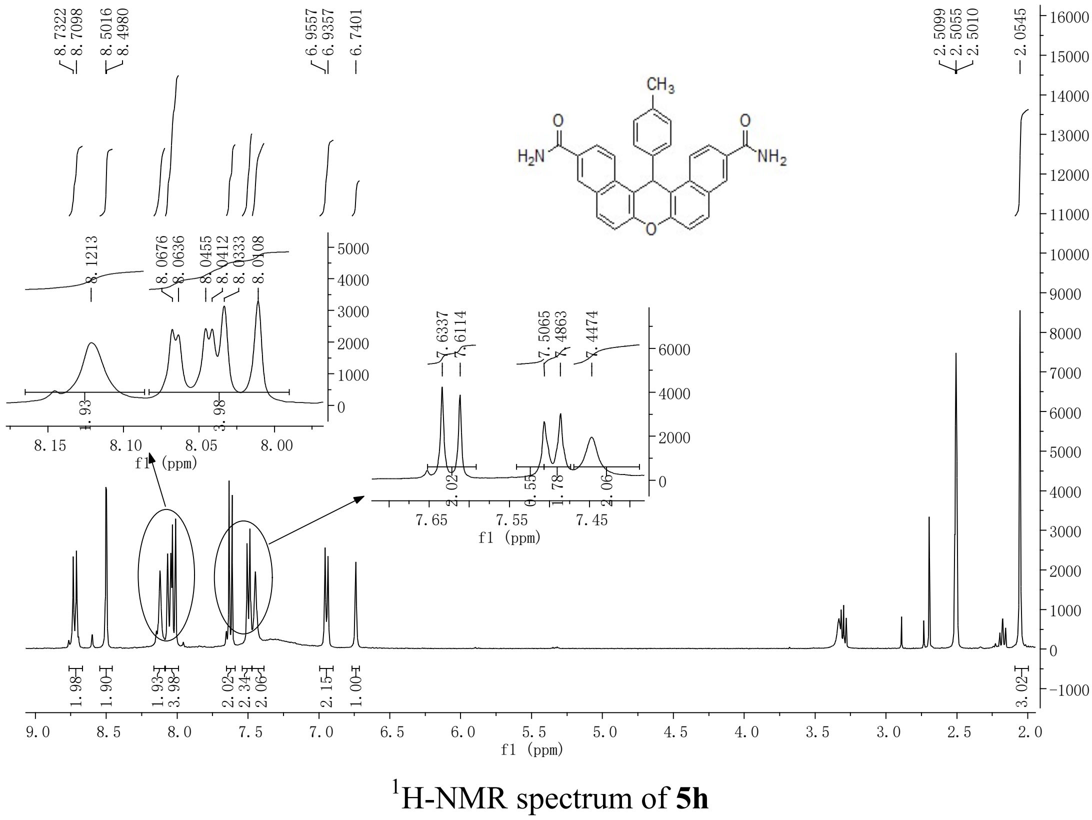

^{1}H-NMR spectrum of **5h**

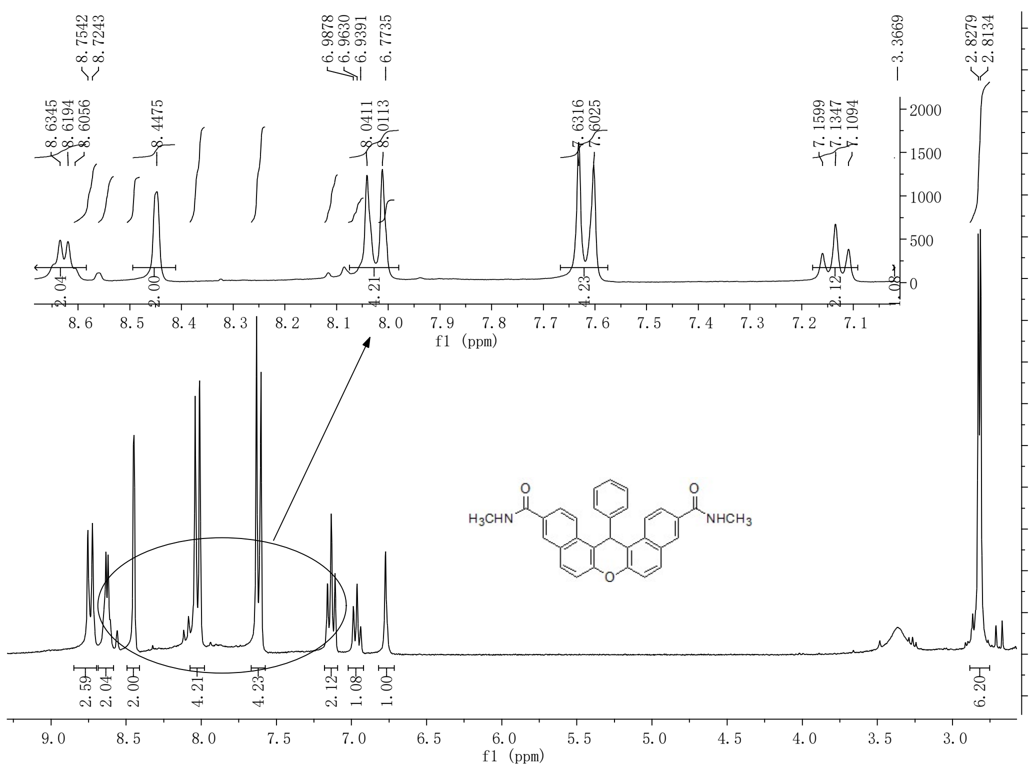

^{1}H-NMR spectrum of **6a**

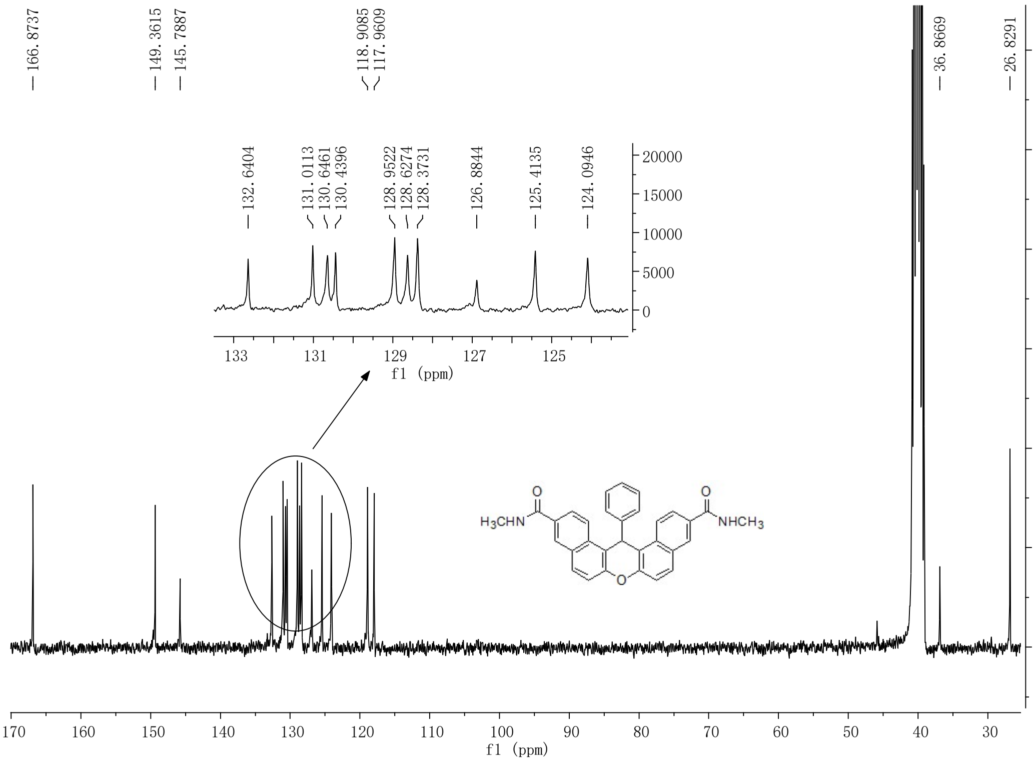

^{13}C-NMR spectrum of **6a**

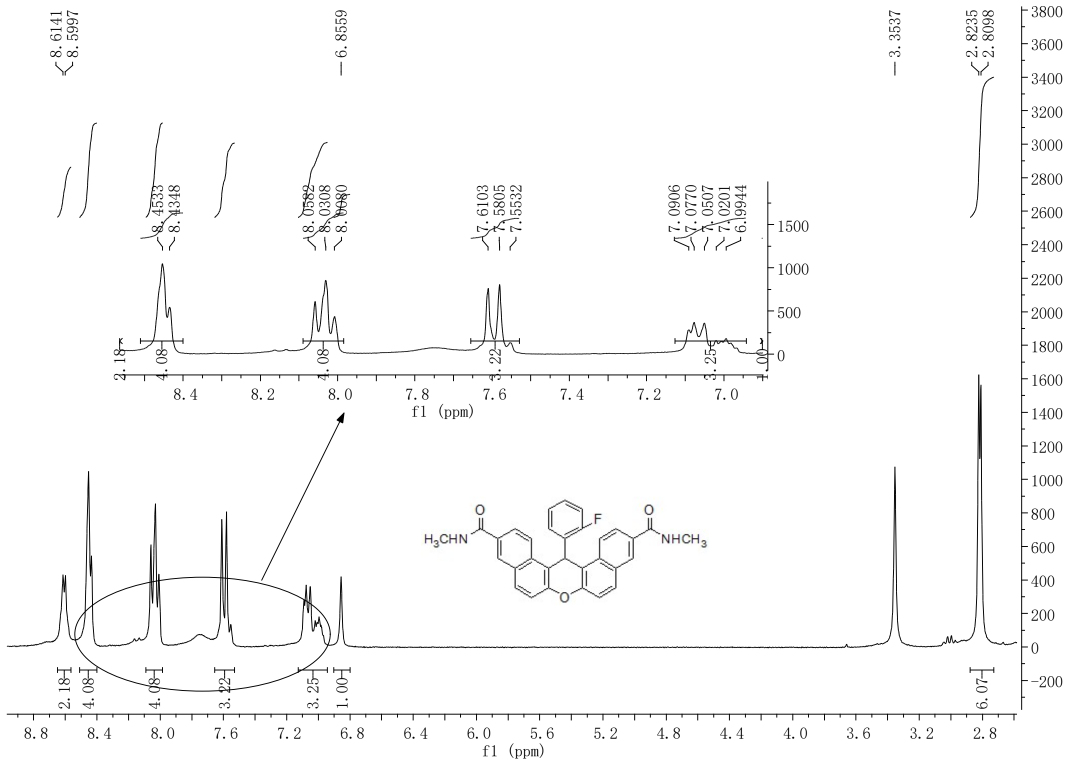

^{1}H-NMR spectrum of **6b**

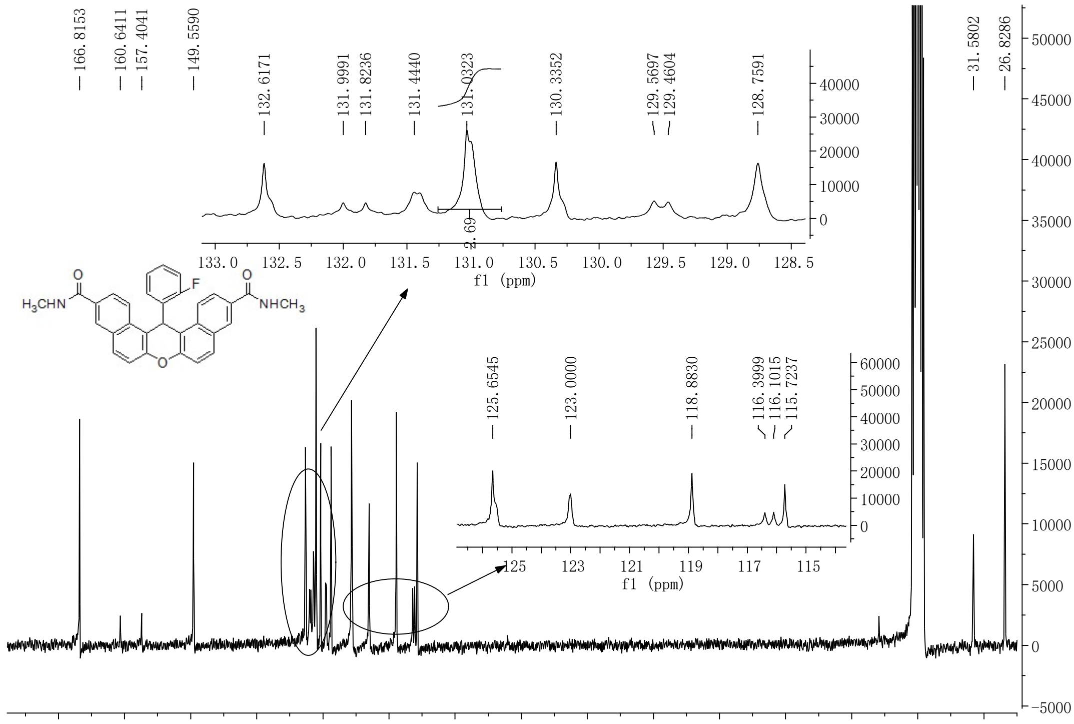

^{13}C-NMR spectrum of **6b**

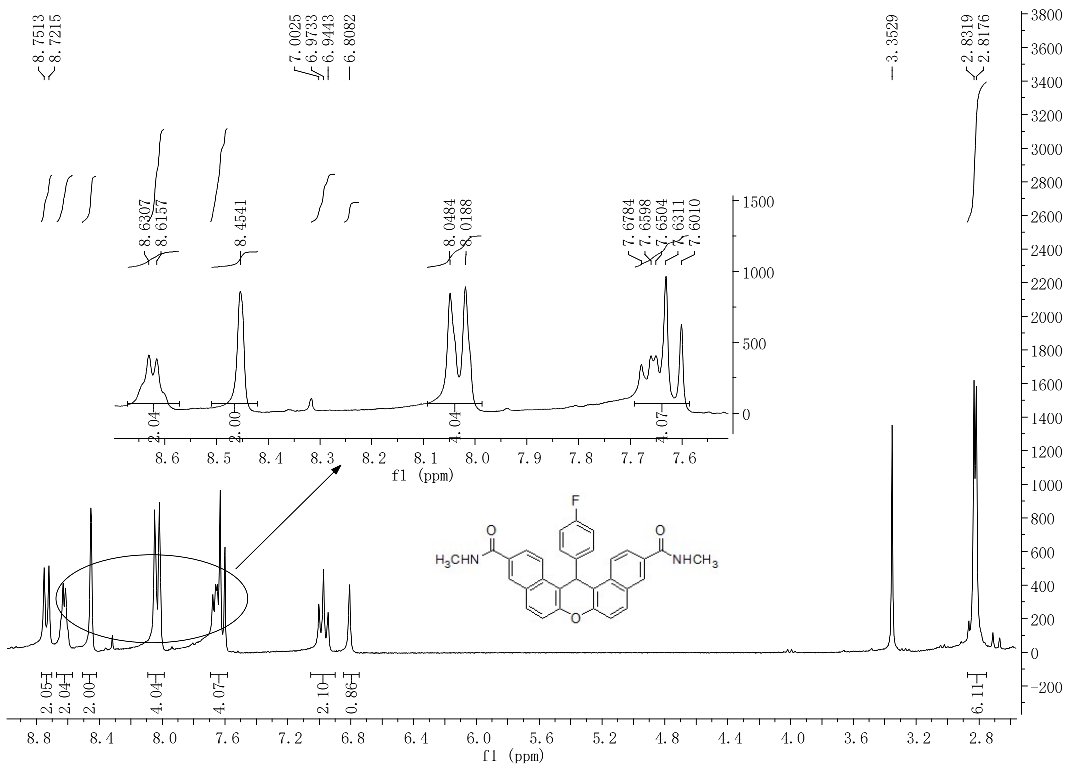

^{1}H-NMR spectrum of **6c**

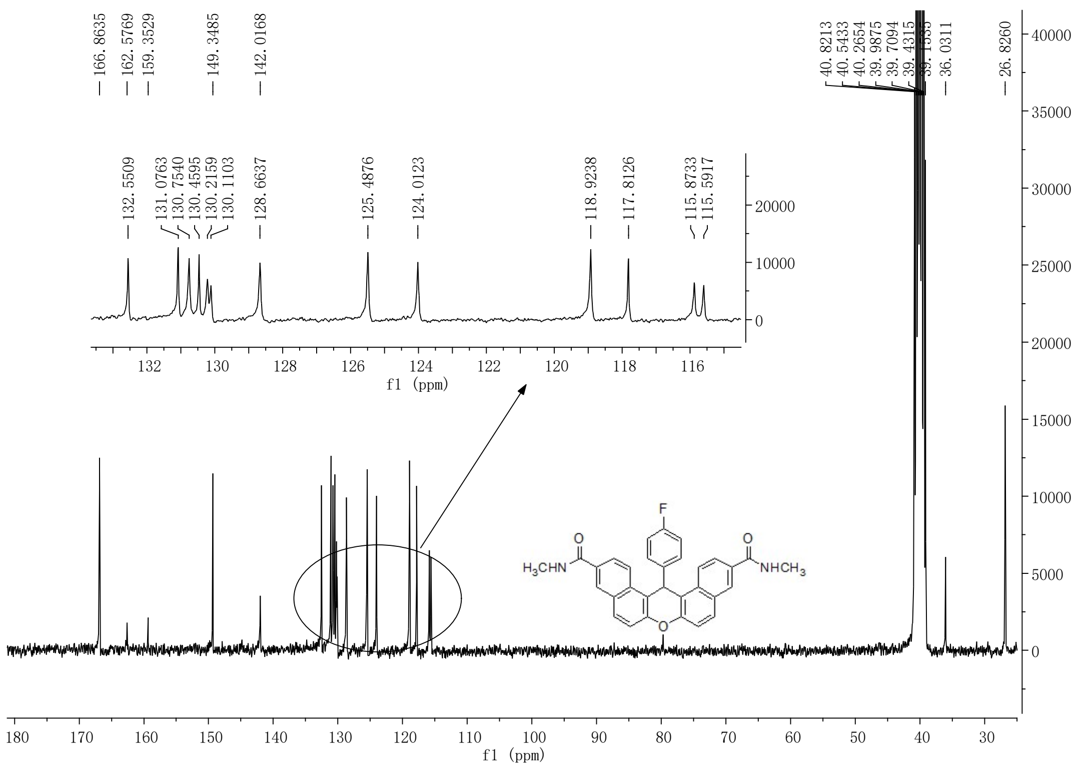

^{13}C-NMR spectrum of **6c**

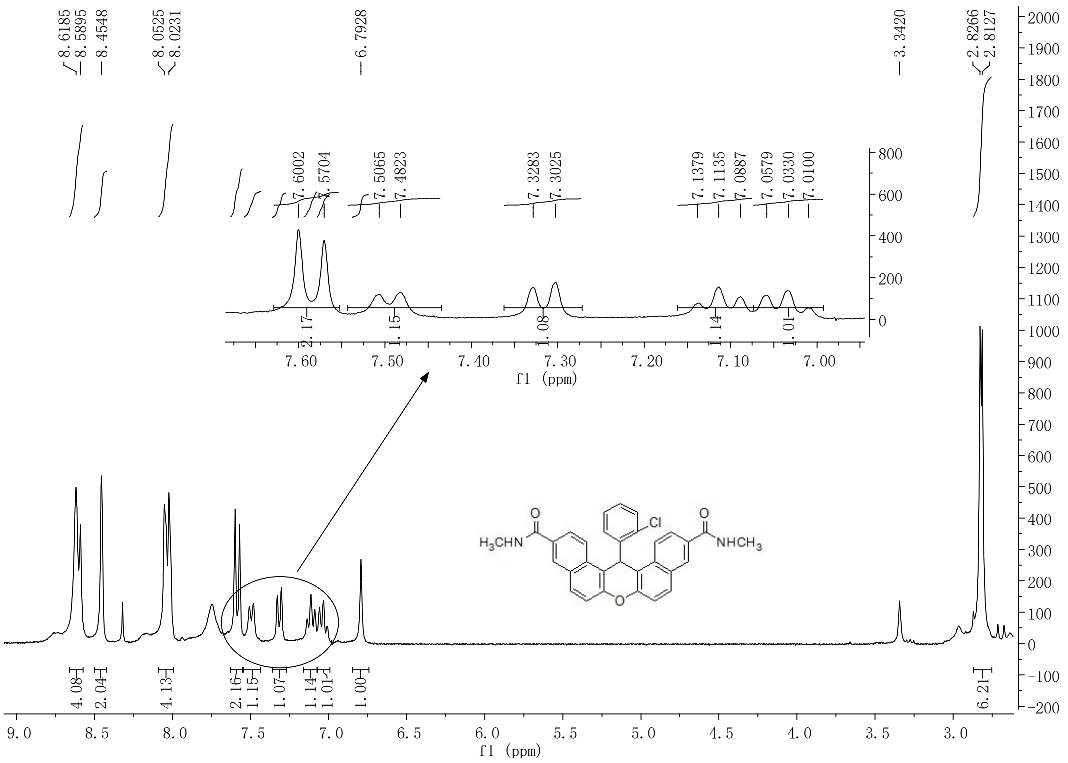

^{1}H-NMR spectrum of **6d**

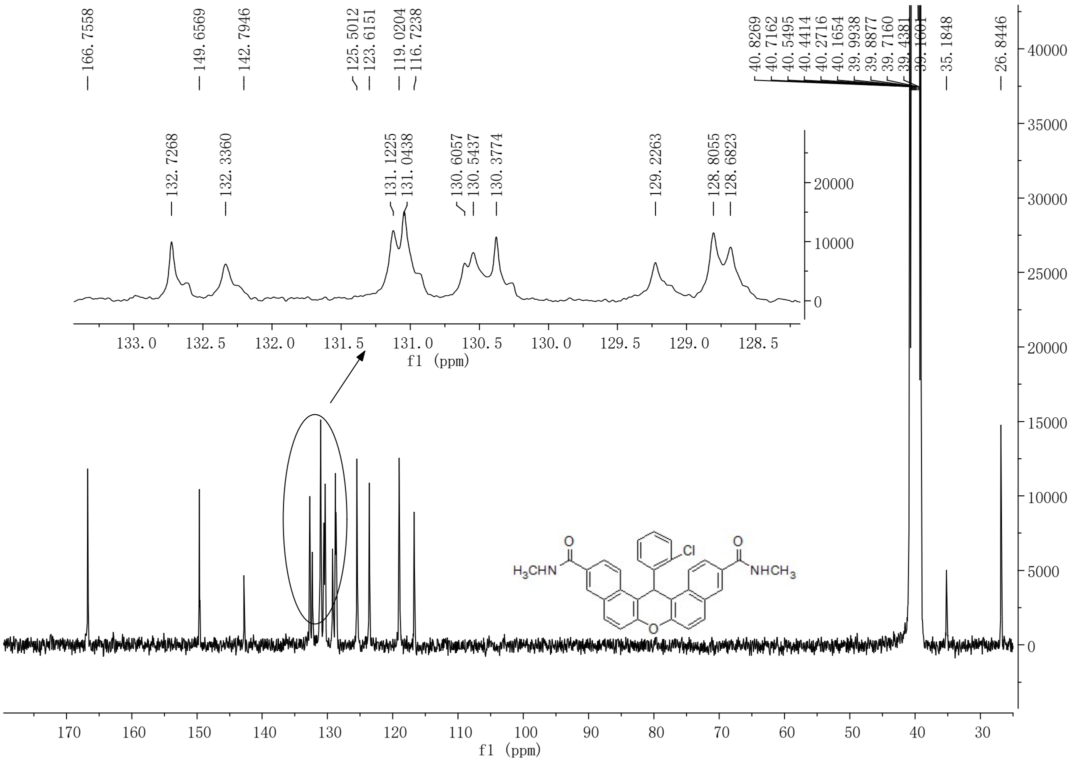

^{13}C-NMR spectrum of **6d**

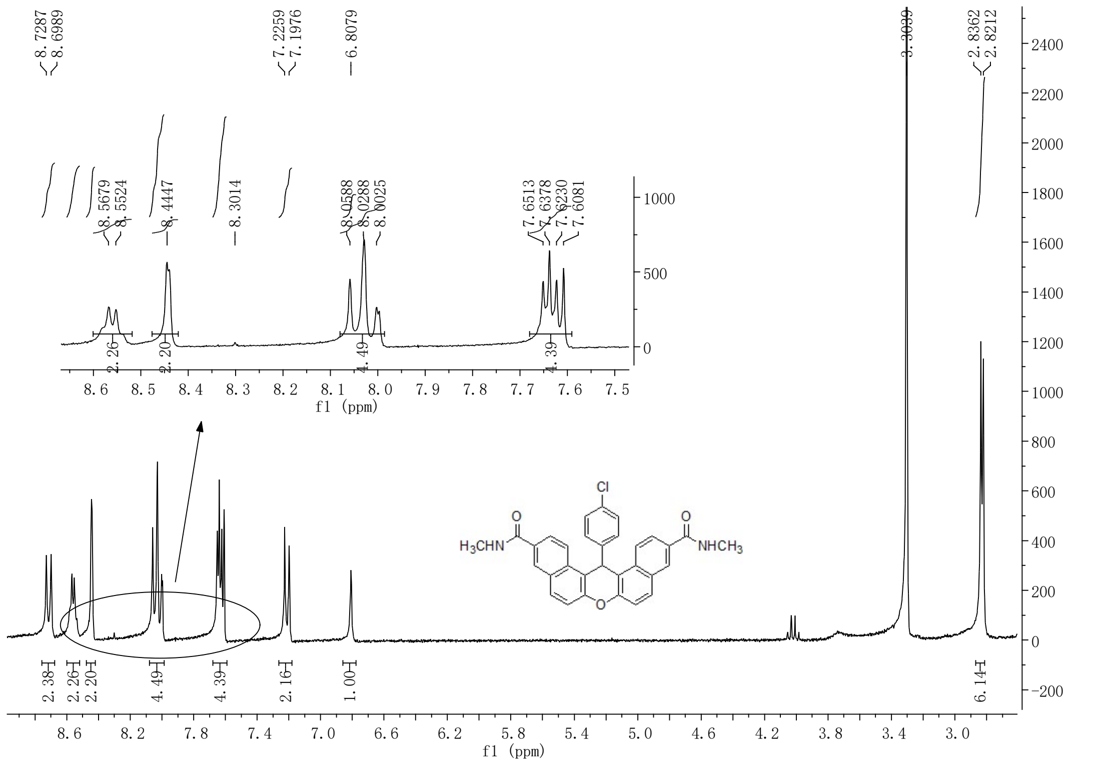

^{1}H-NMR spectrum of **6e**

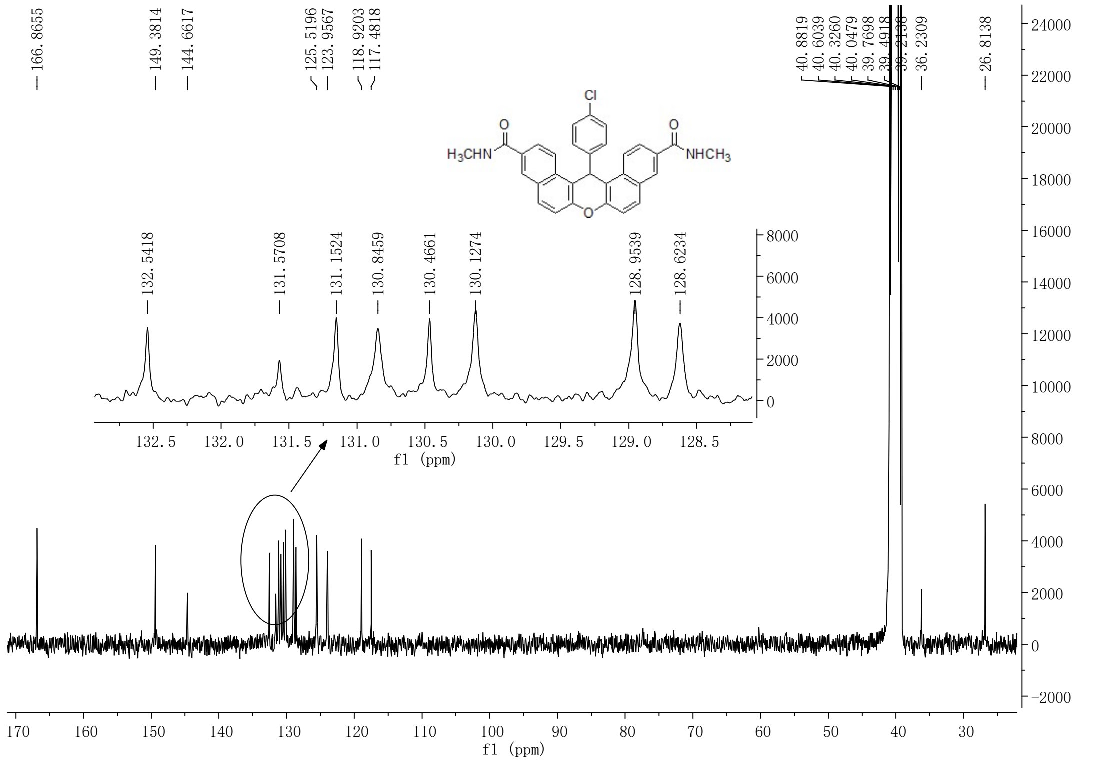

^{13}C-NMR spectrum of **6e**

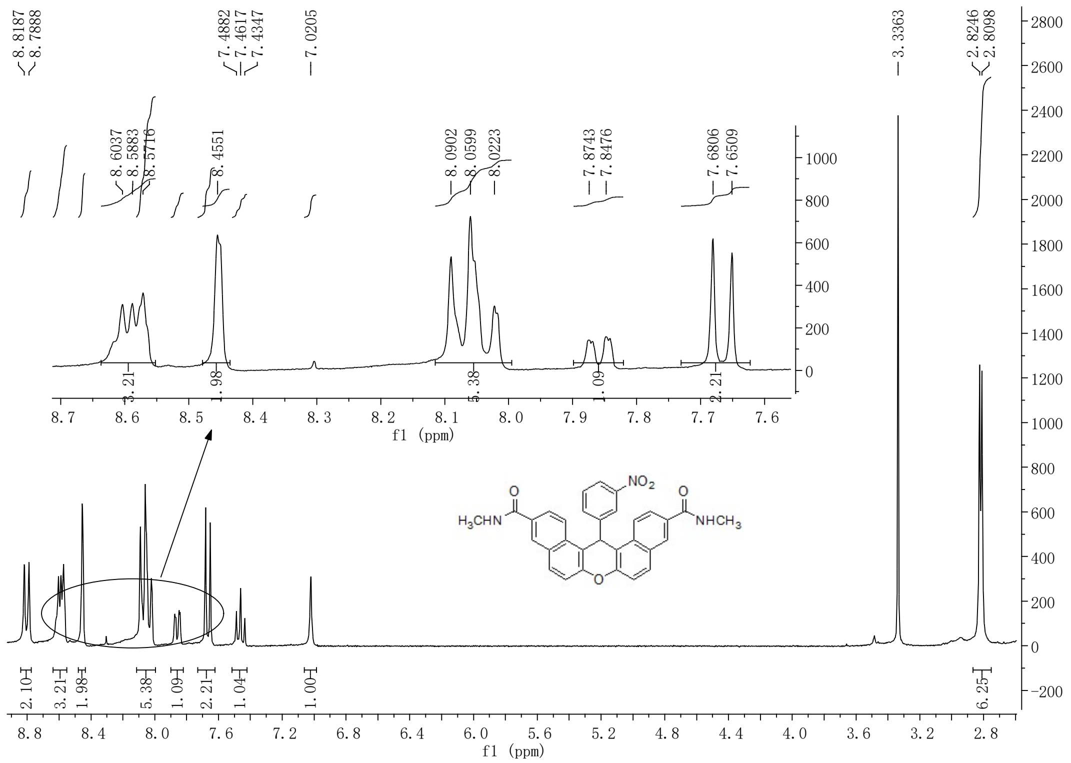

^{1}H-NMR spectrum of **6f**

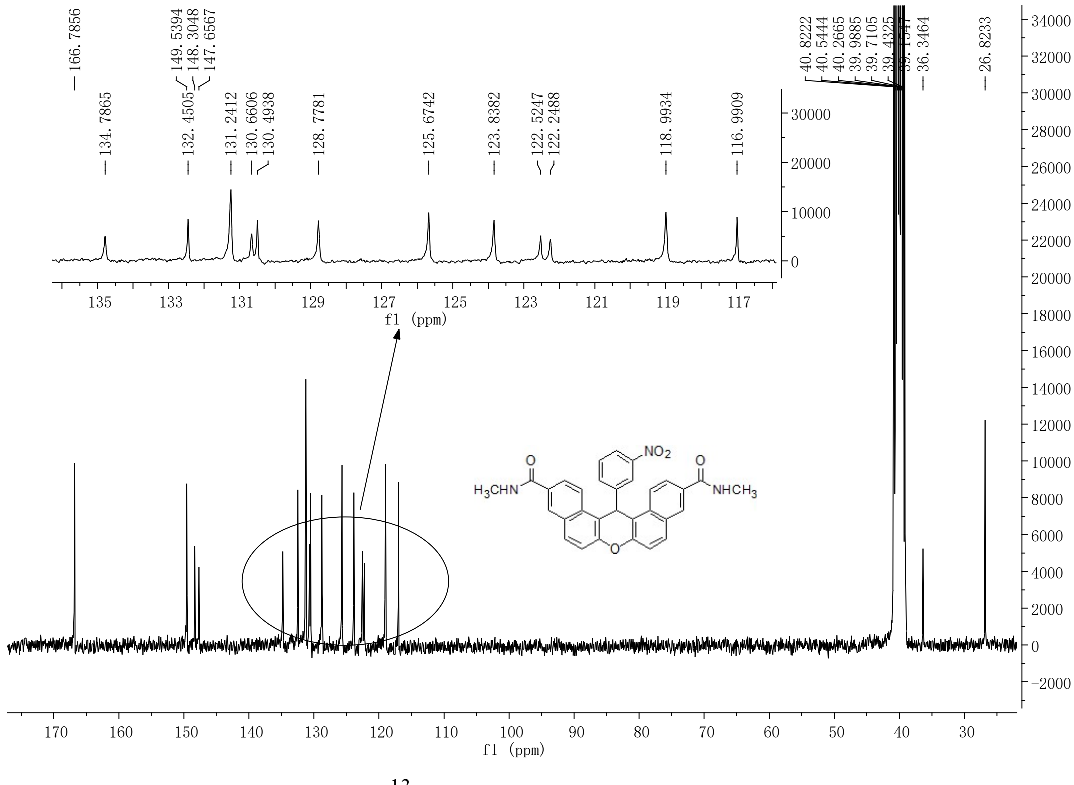

^{13}C-NMR spectrum of **6f**

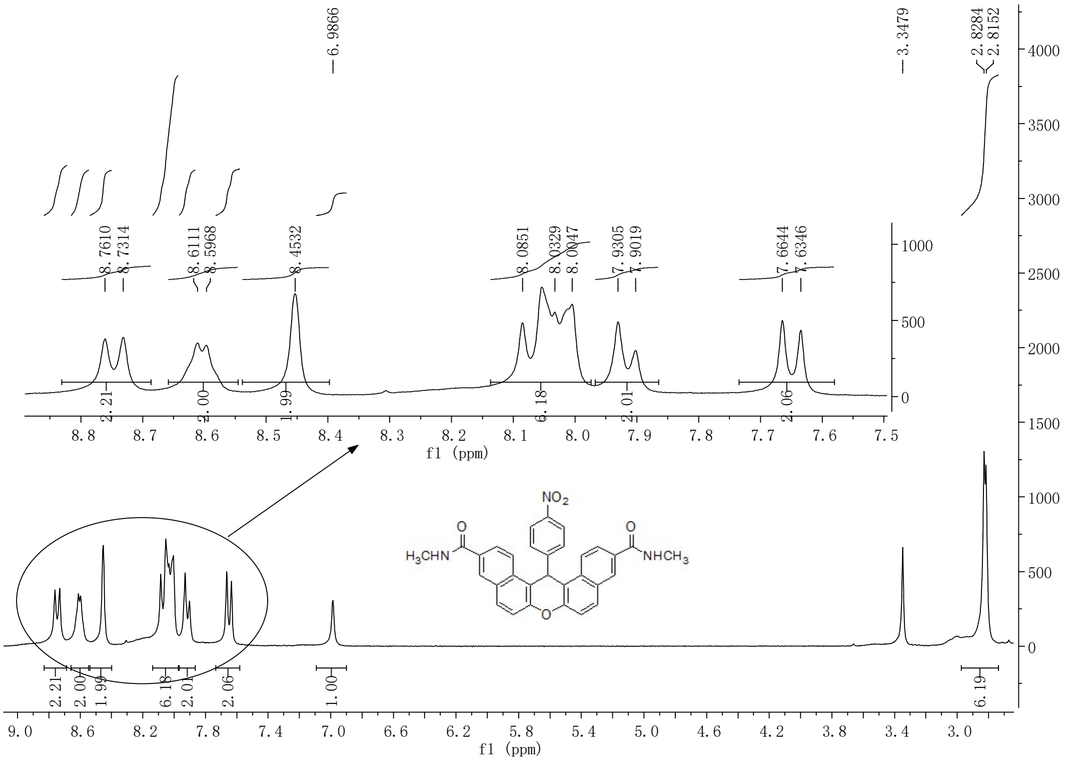

^{1}H-NMR spectrum of **6g**

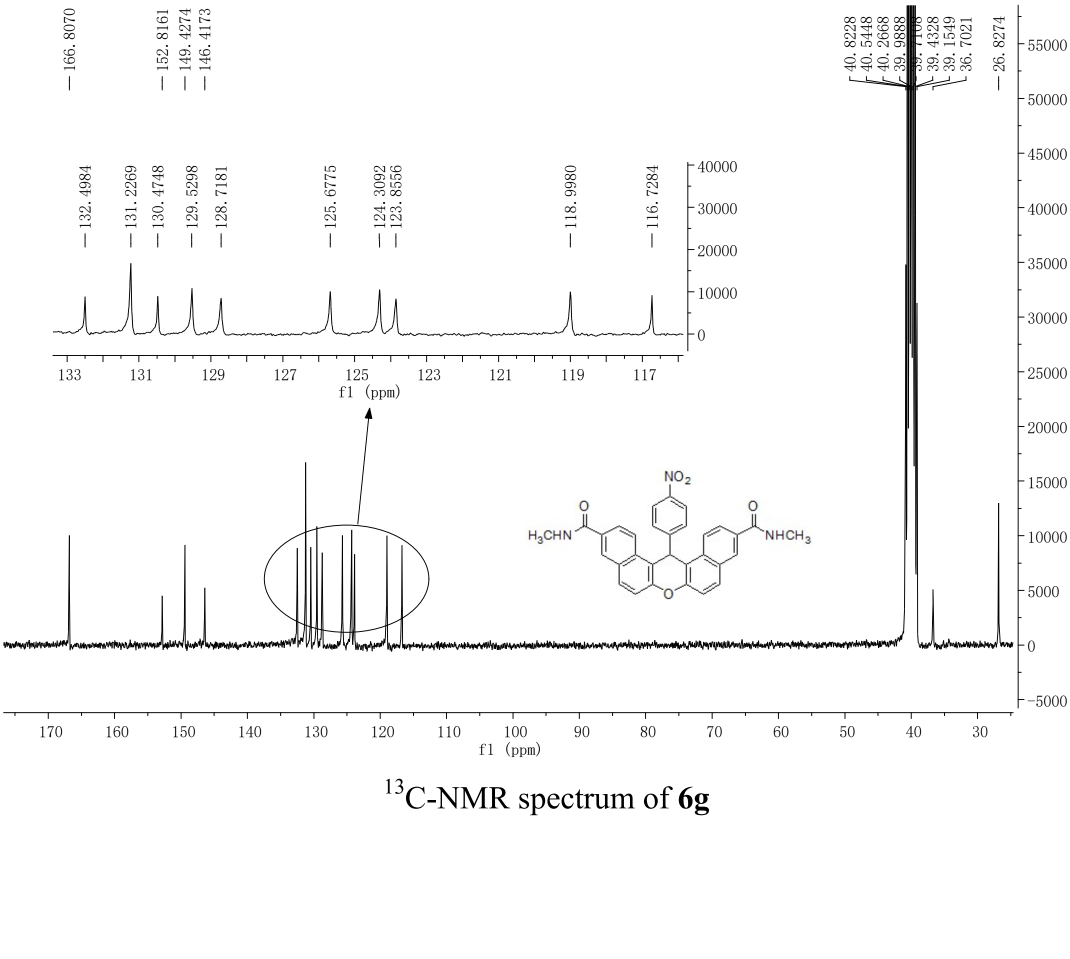

^{13}C-NMR spectrum of **6g**

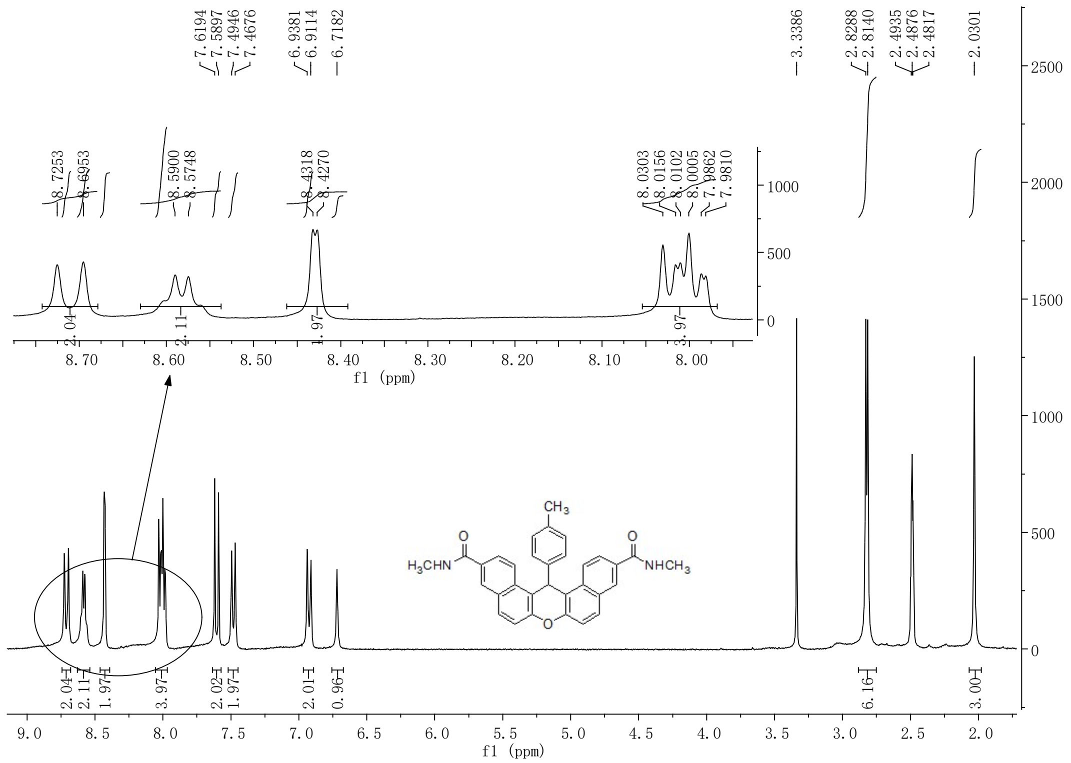

^{1}H-NMR spectrum of **6h**

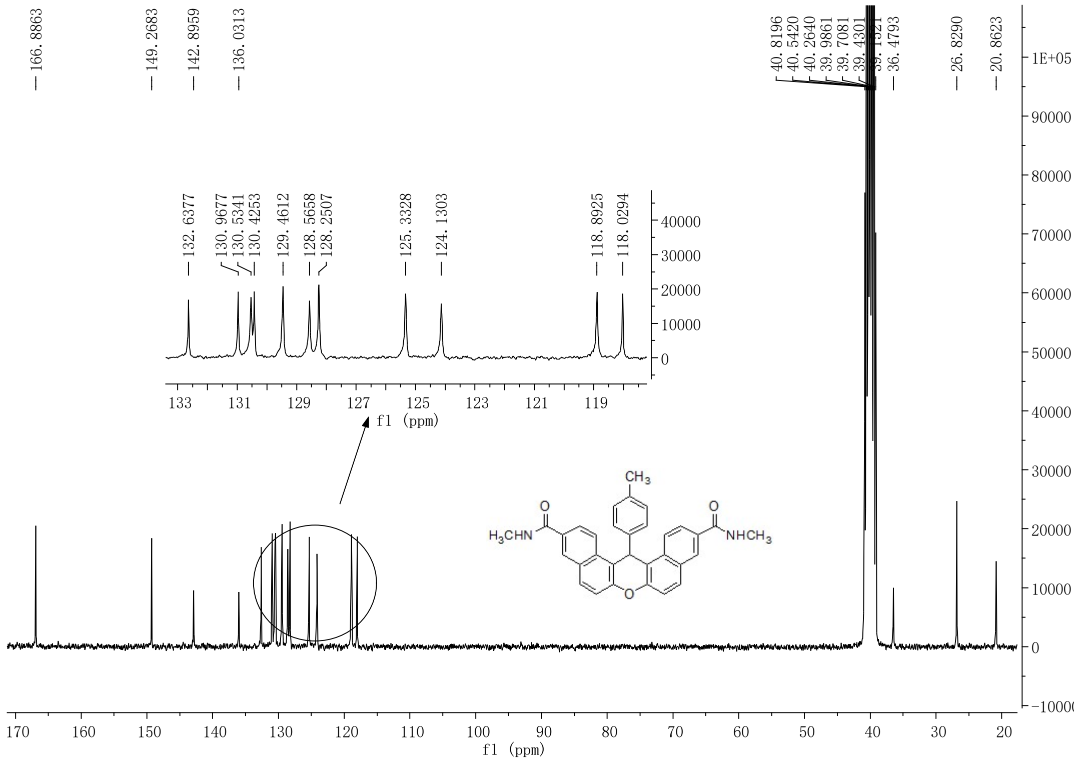

^{13}C-NMR spectrum of **6h**

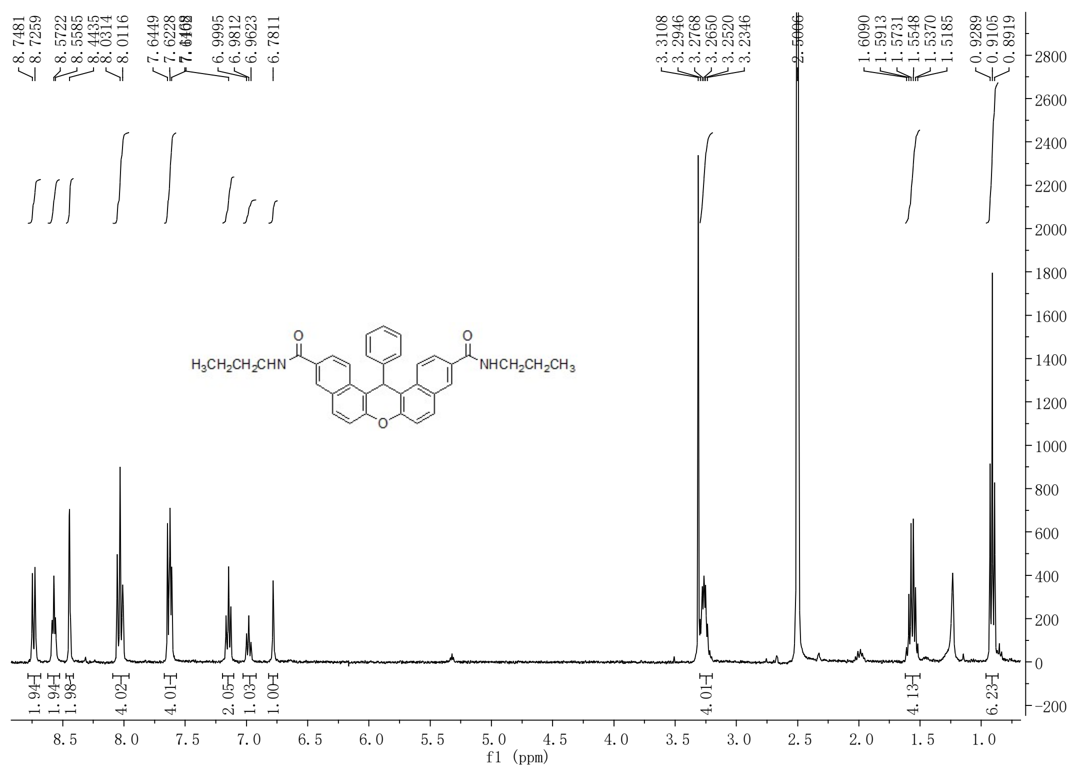

^{1}H-NMR spectrum of **7a**

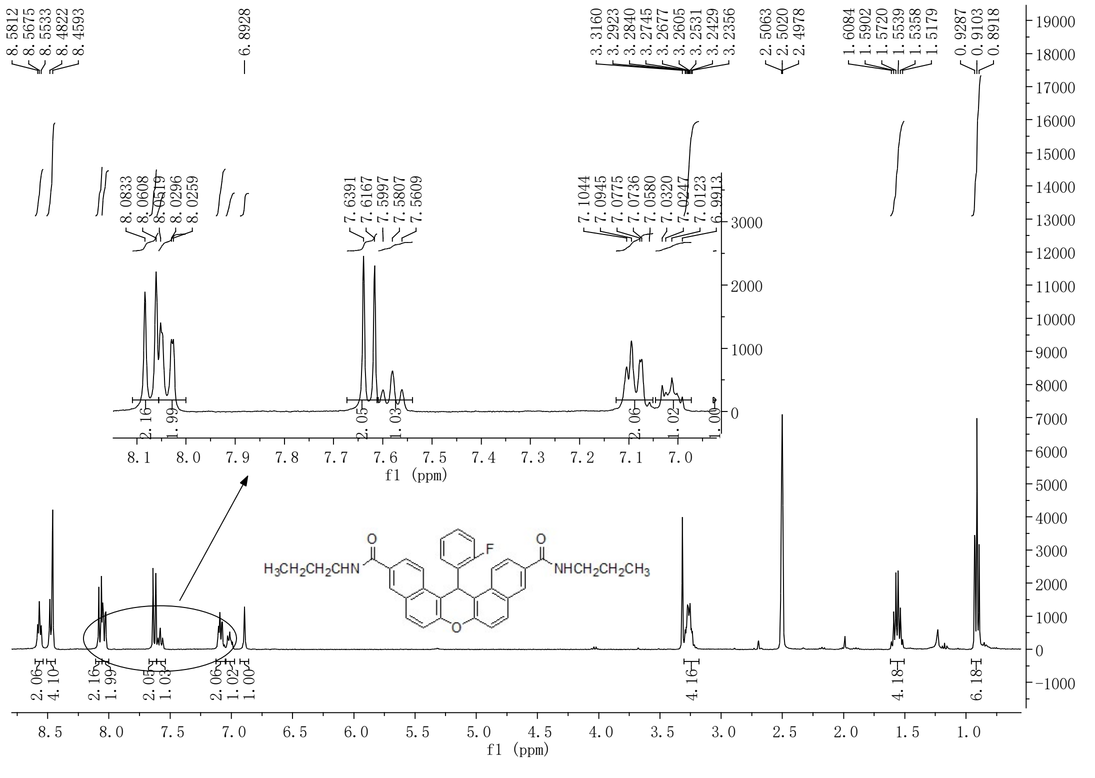

^{1}H-NMR spectrum of **7b**

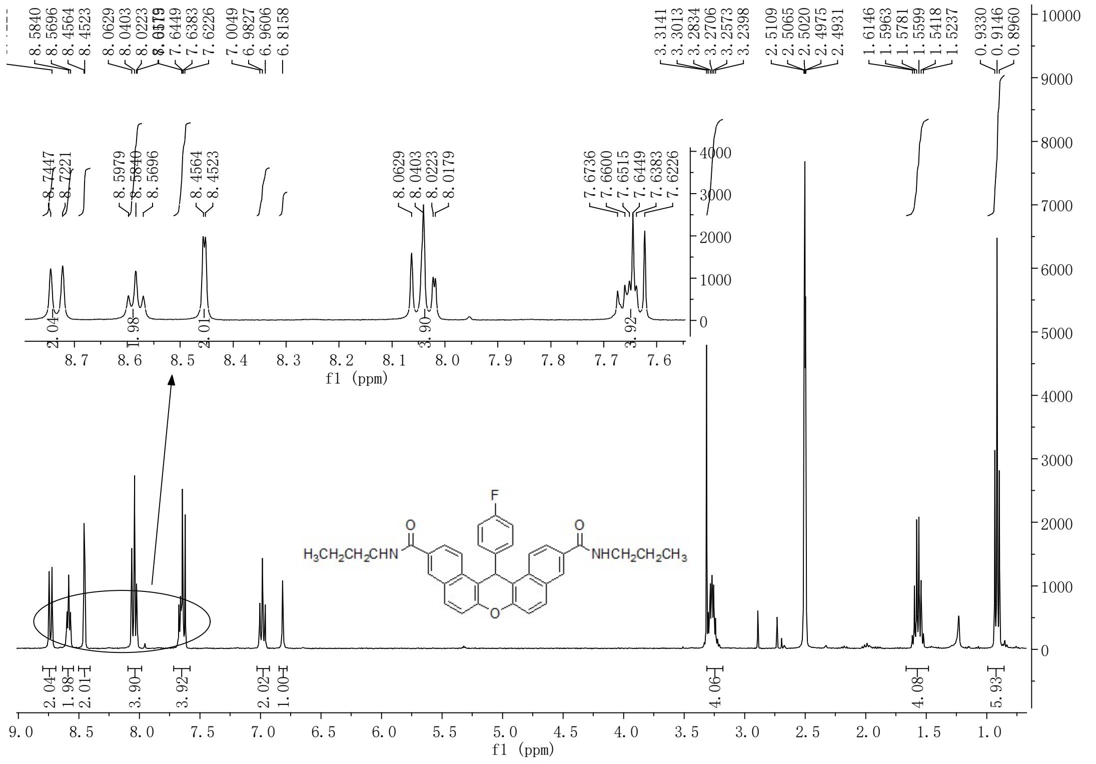

^{1}H-NMR spectrum of **7c**

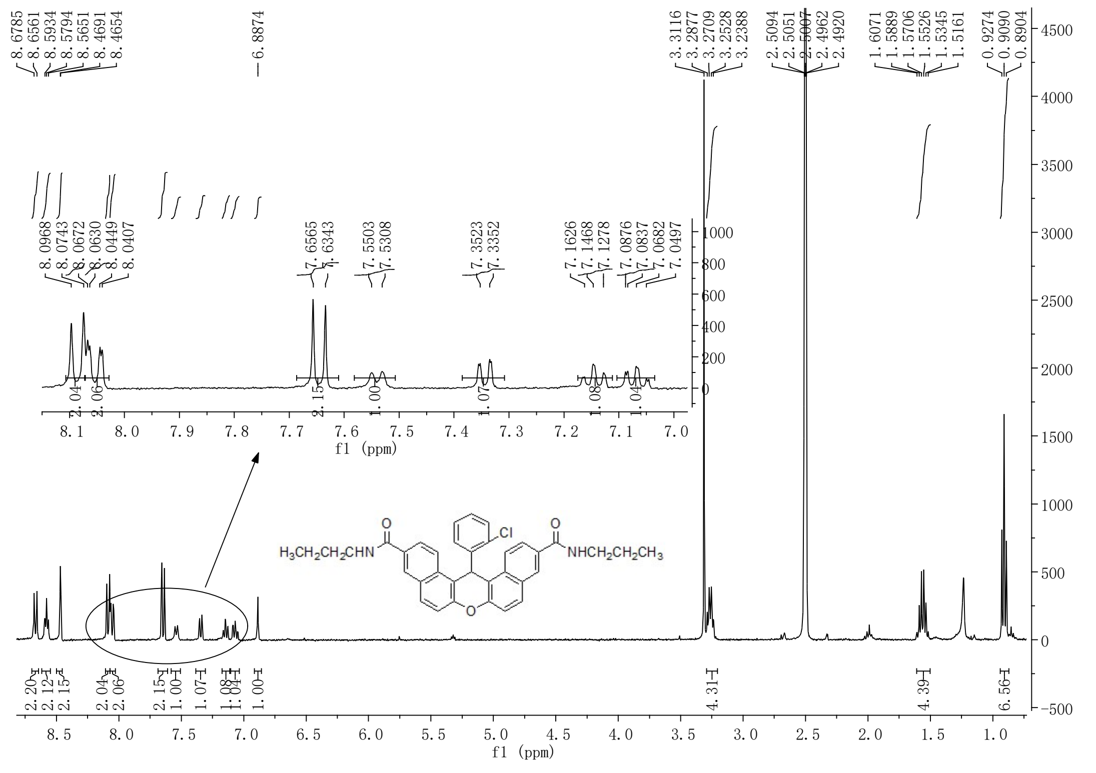

^{1}H-NMR spectrum of **7d**

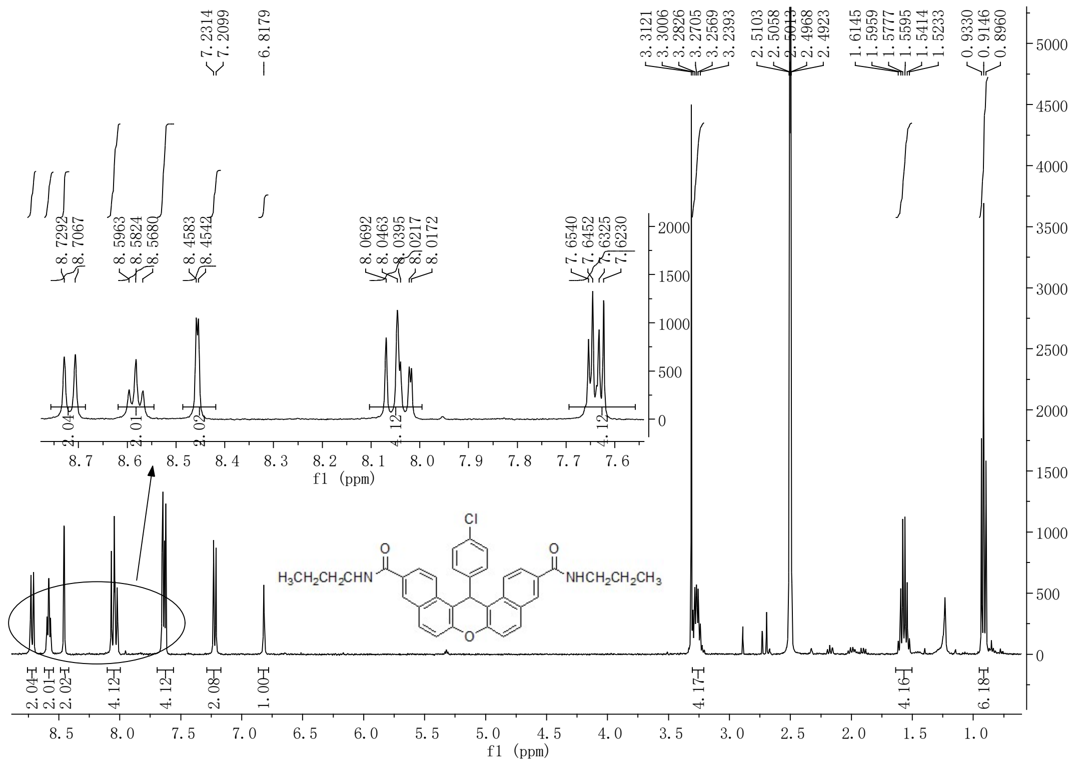

^{1}H-NMR spectrum of **7e**

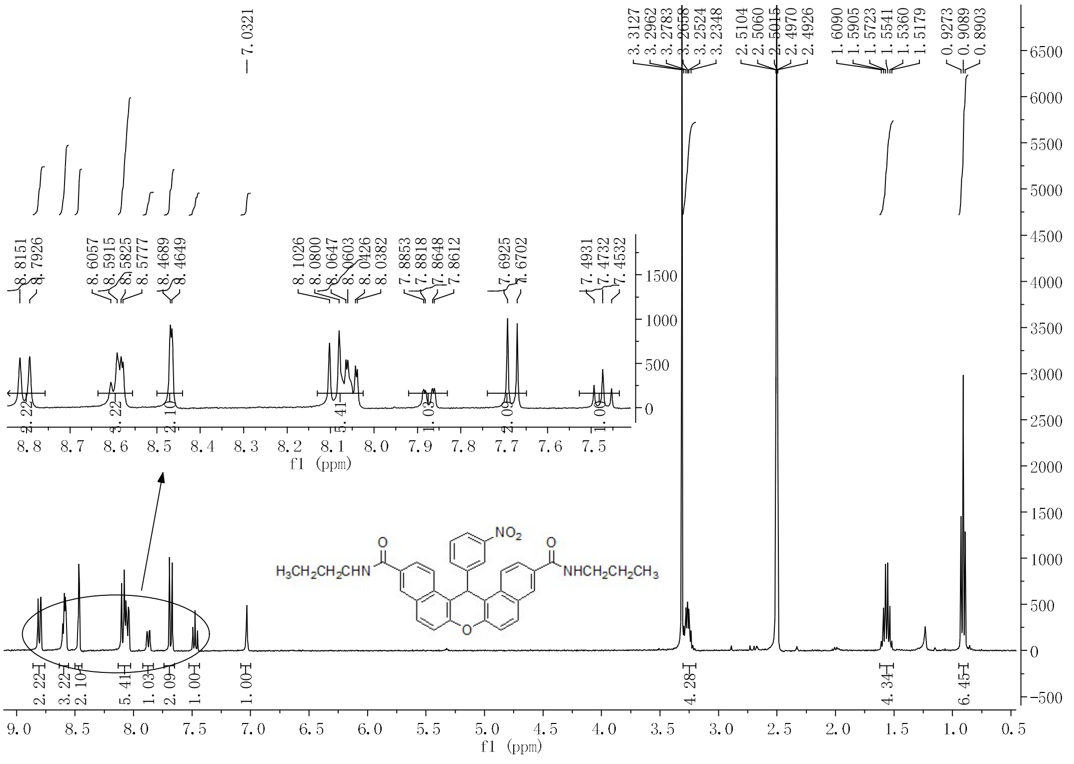

^{1}H-NMR spectrum of **7f**

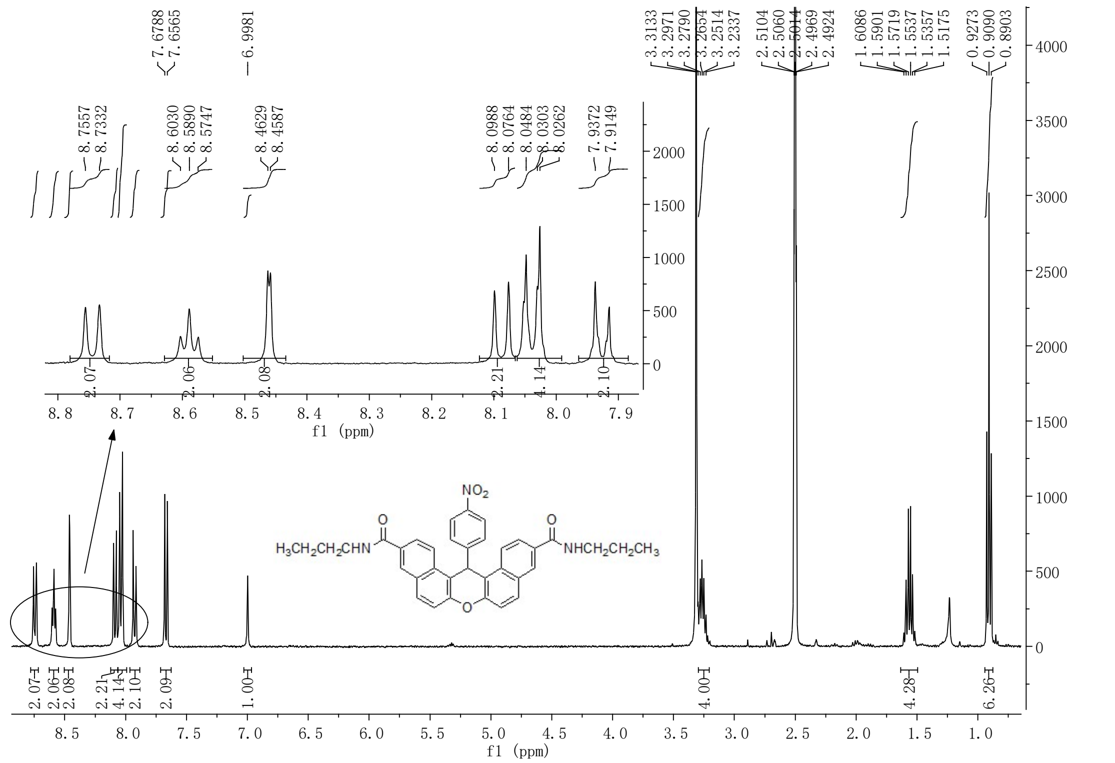

^{1}H-NMR spectrum of **7g**

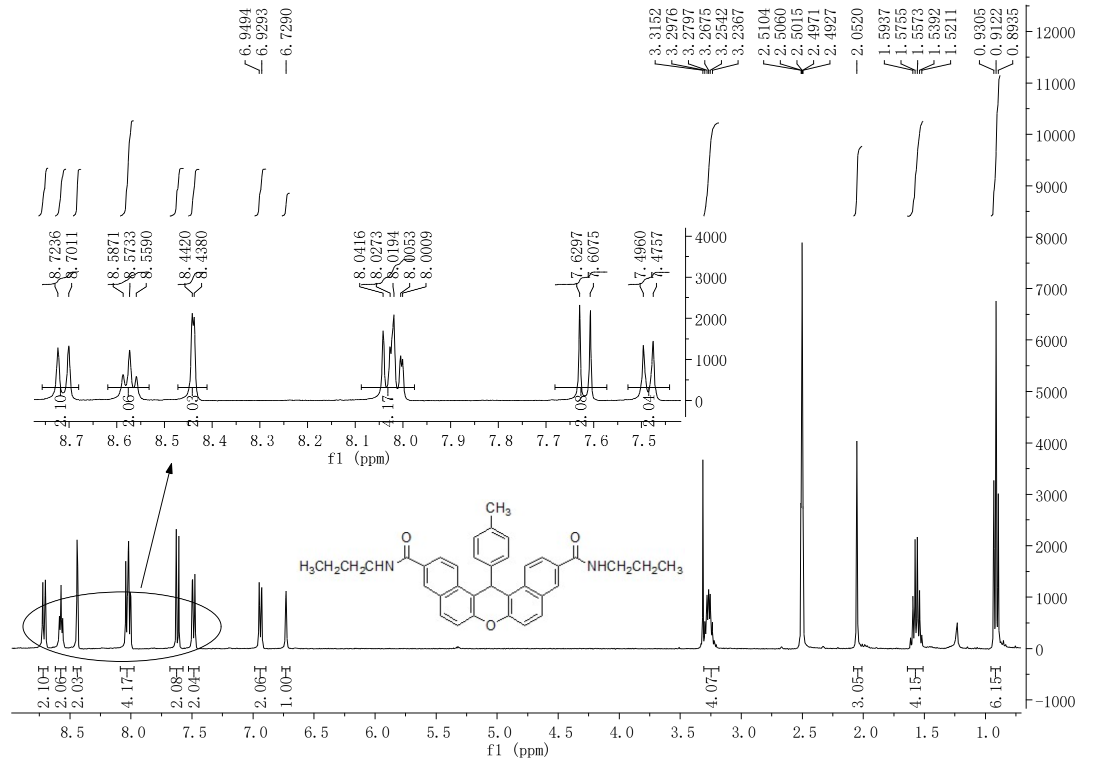

^{1}H-NMR spectrum of **7h**

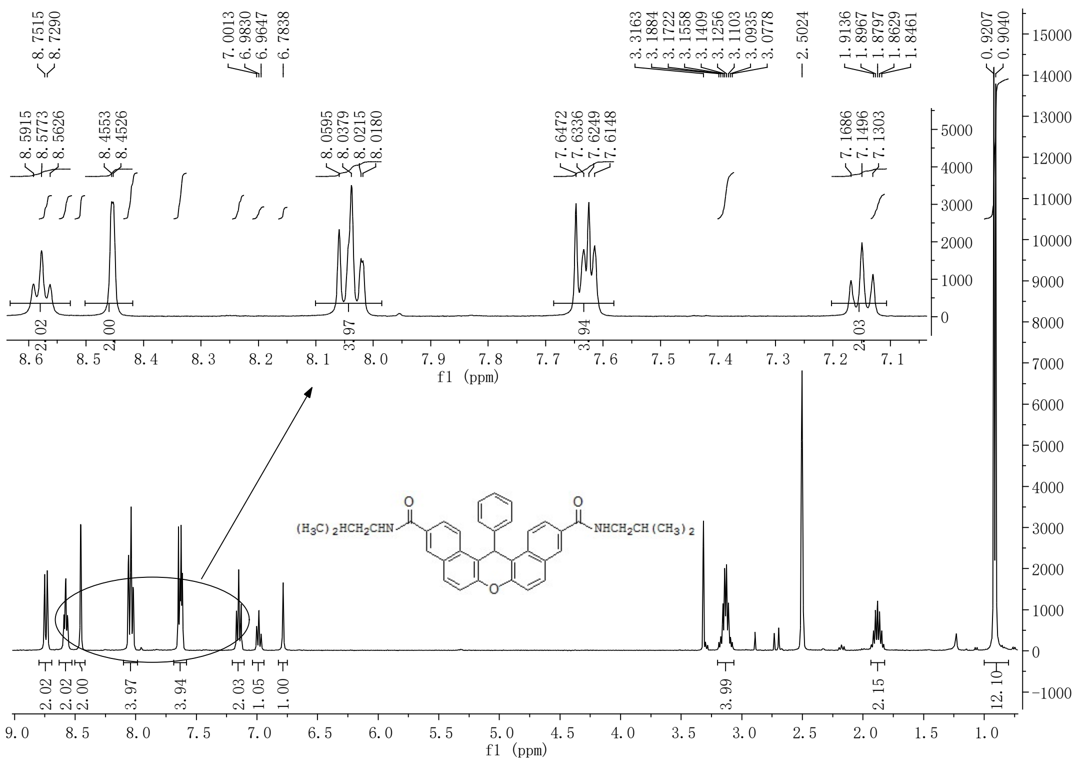

^{1}H-NMR spectrum of **8a**

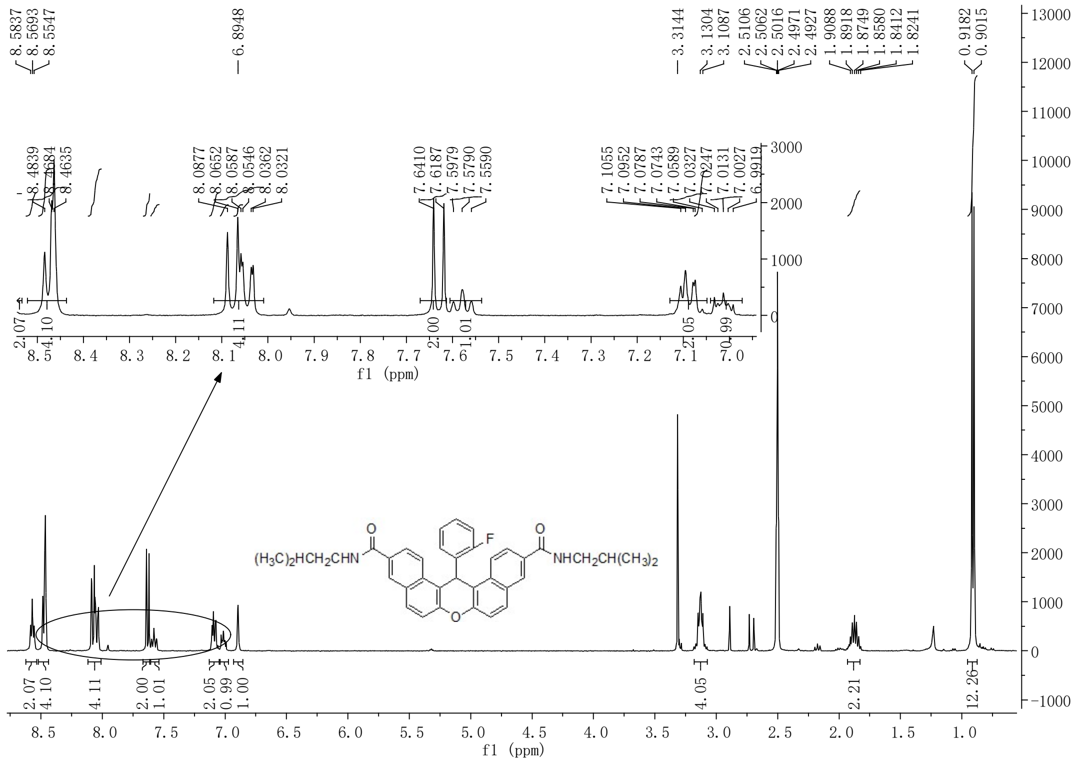

^{1}H-NMR spectrum of **8b**

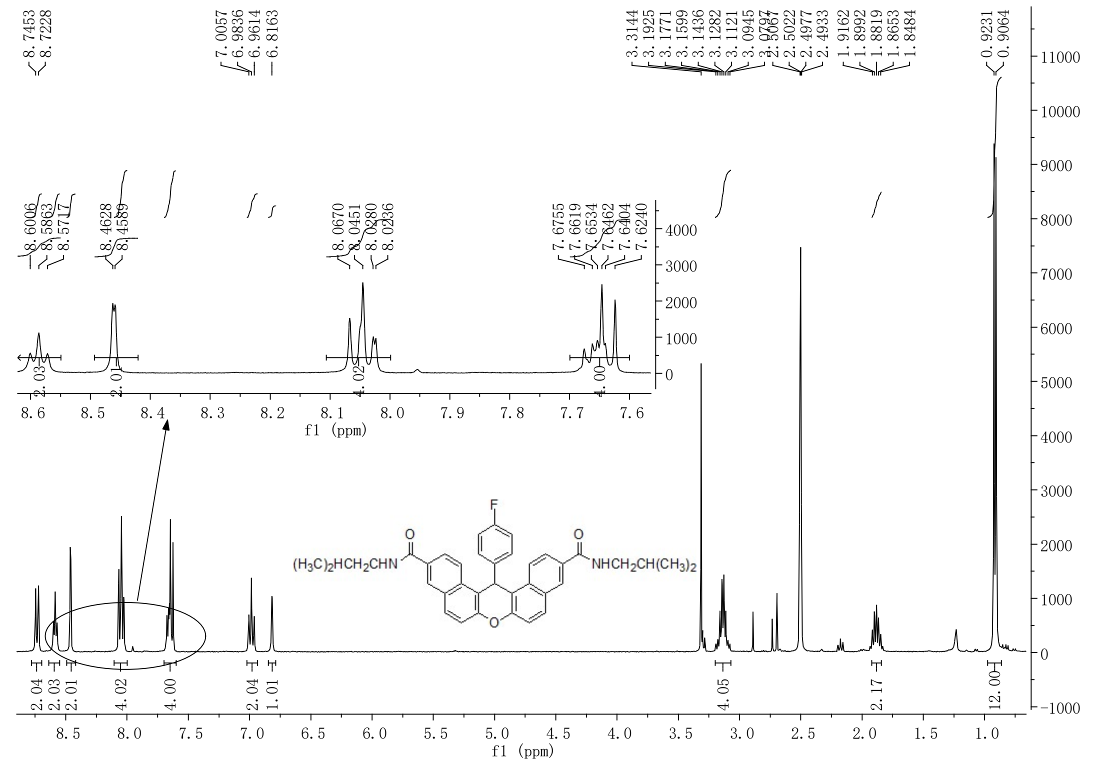

^{1}H-NMR spectrum of **8c**

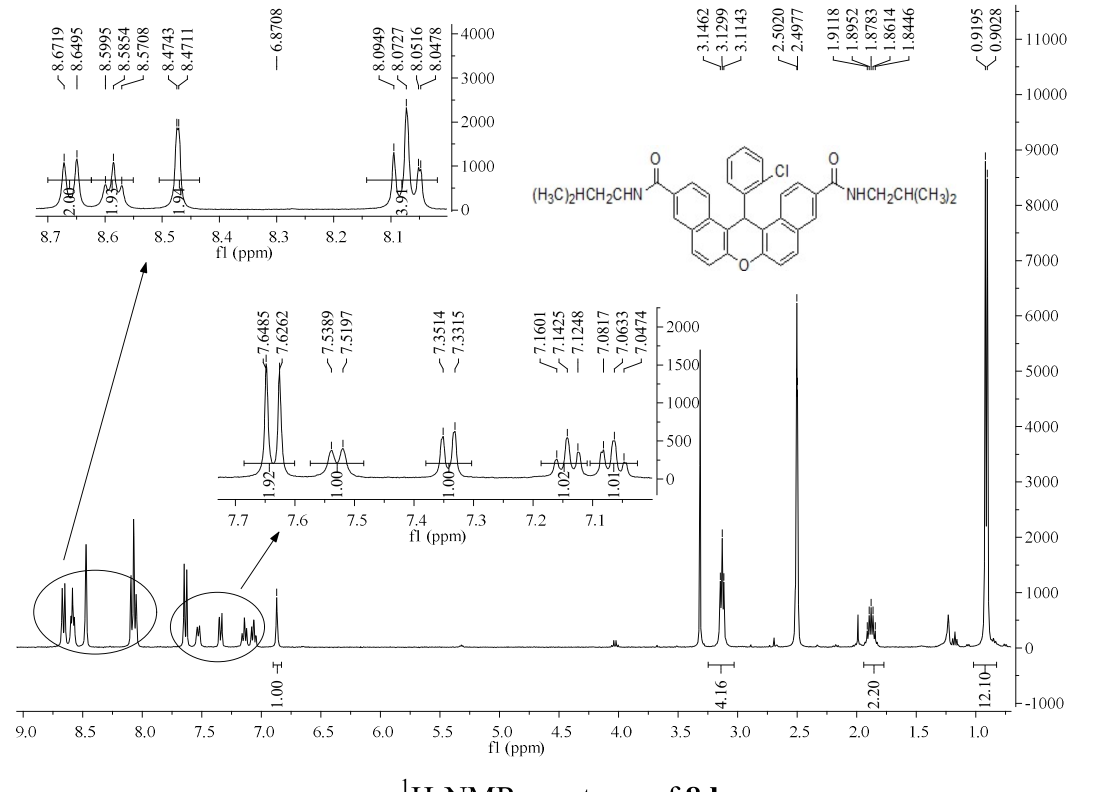

^{1}H-NMR spectrum of **8d**

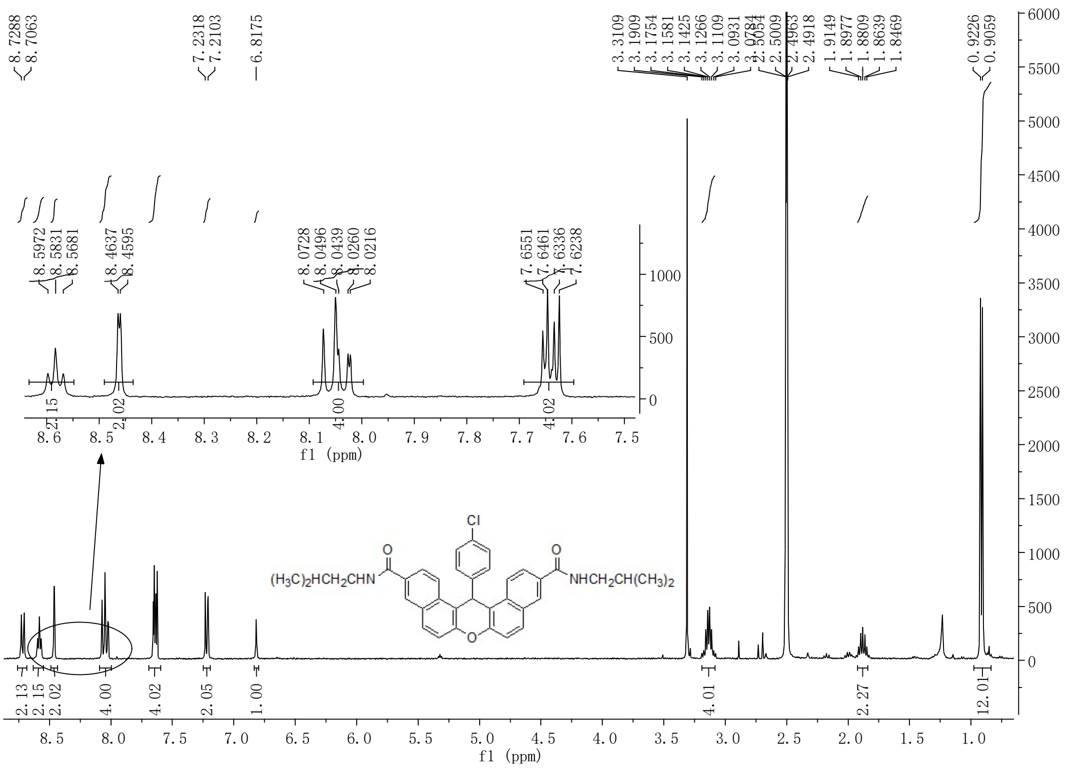

^{1}H-NMR spectrum of **8e**

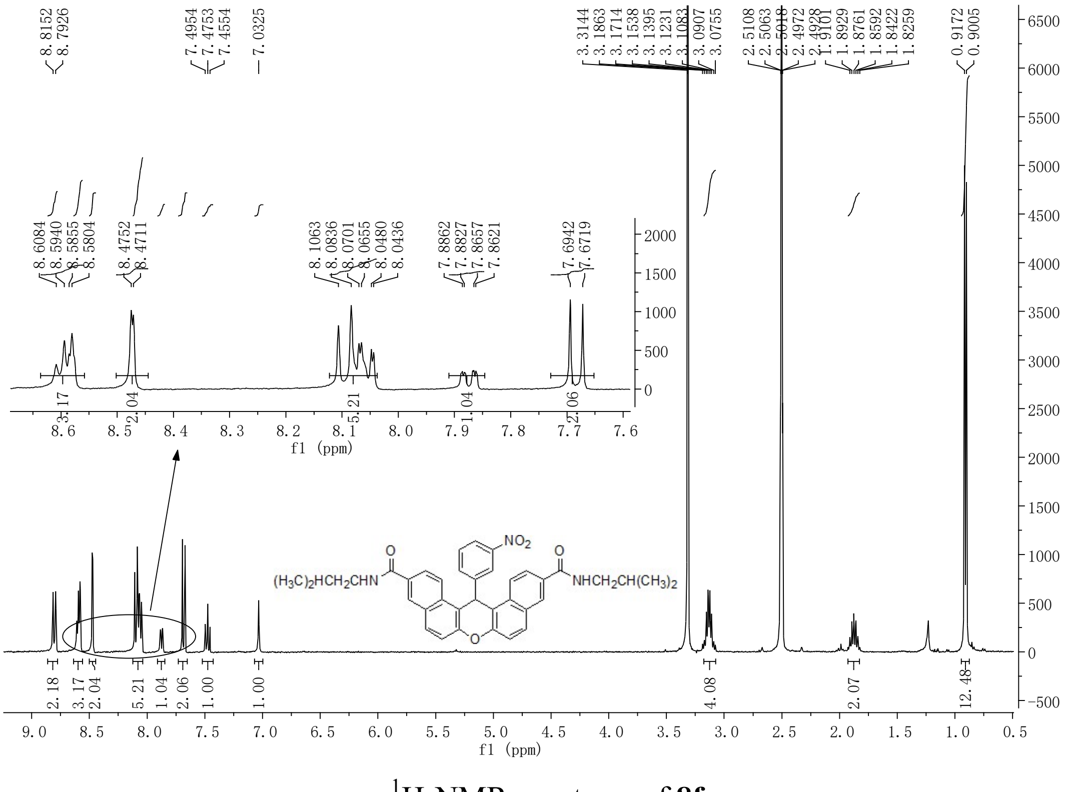

^{1}H-NMR spectrum of **8f**

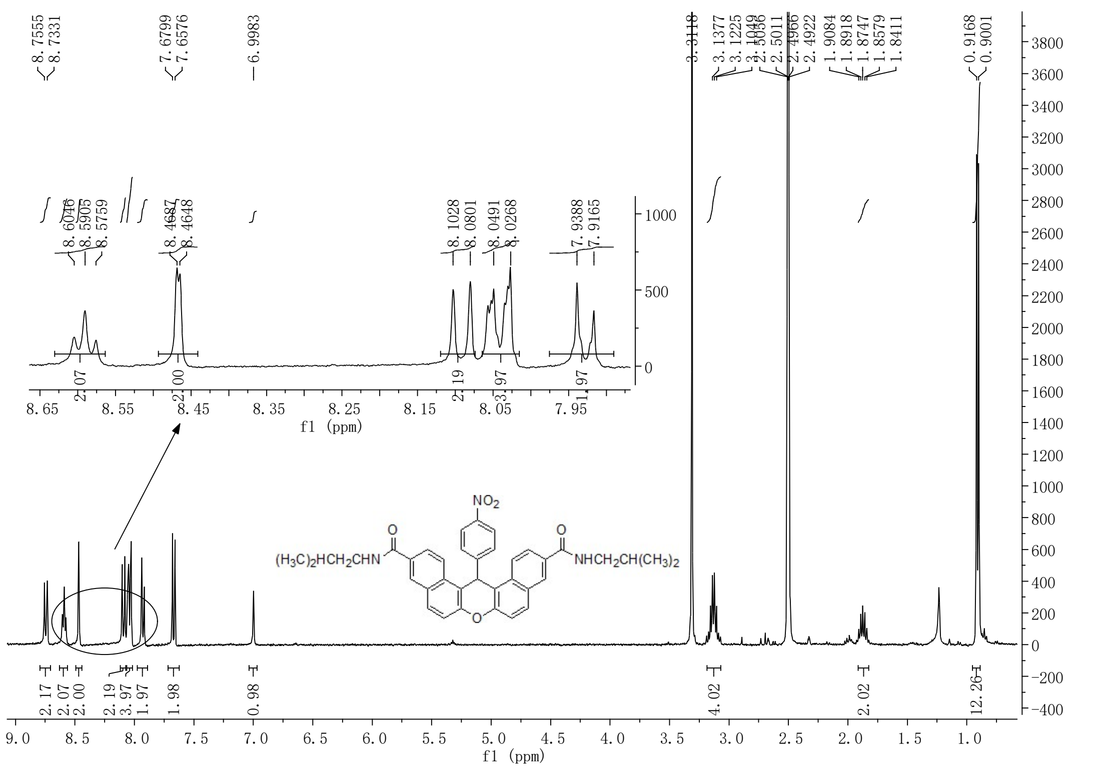

^{1}H-NMR spectrum of **8g**

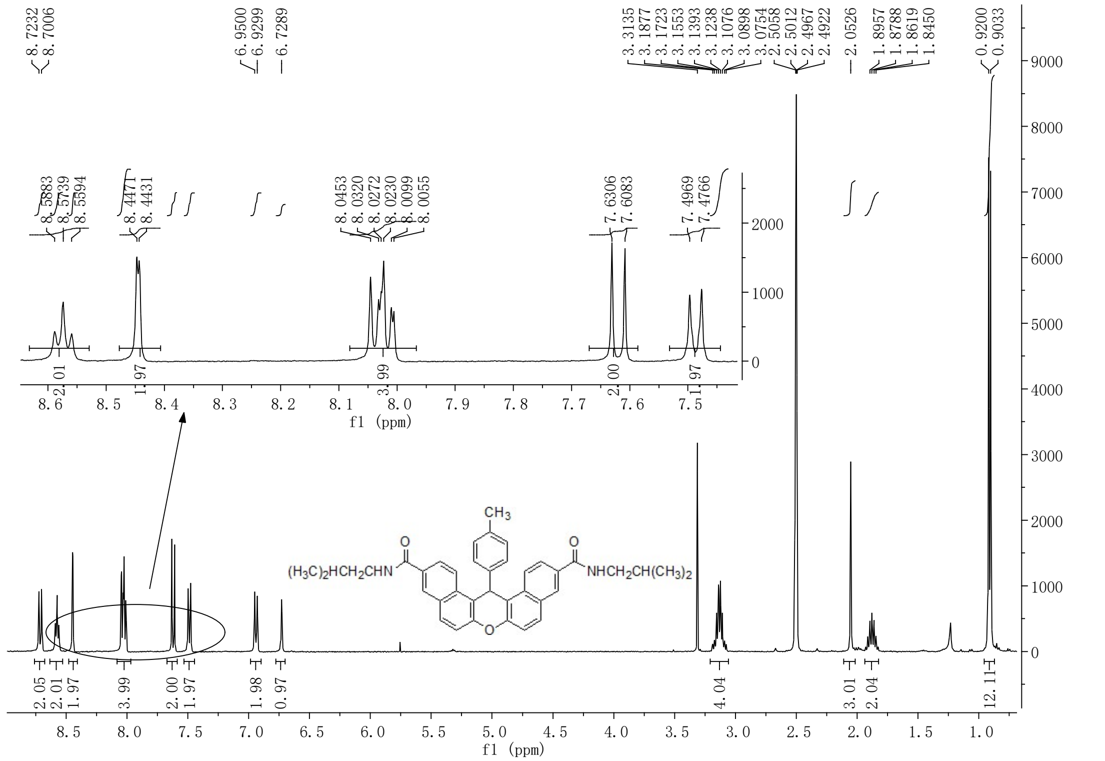

^{1}H-NMR spectrum of **8h**
